# A revision of malbranchea-like fungi from clinical specimens in the United States of America reveals unexpected novelty

**DOI:** 10.1186/s43008-021-00075-x

**Published:** 2021-09-07

**Authors:** Ernesto Rodríguez-Andrade, José F. Cano-Lira, Nathan Wiederhold, Alba Pérez-Cantero, Josep Guarro, Alberto M. Stchigel

**Affiliations:** 1 Mycology Unit, Medical School, Universitat Rovira i Virgili (URV), Sant Llorenç 21, 43201 Reus, Tarragona Spain; 2 Fungus Testing Laboratory, University of Texas Health Science Center, San Antonio, TX USA

**Keywords:** Antifungals, *Arachnomycetales*, *Auxarthron*, Clinical fungi, *Malbranchea*, *Onygenales*, New taxa

## Abstract

The fungi of the order *Onygenales* can cause important human infections; however, their taxonomy and worldwide occurrence is still little known. We have studied and identified a representative number of clinical fungi belonging to that order from a reference laboratory in the USA. A total of 22 strains isolated from respiratory tract (40%) and human skin and nails (27.2%) showed a malbranchea-like morphology. Six genera were phenotypically and molecularly identified, i.e. *Auxarthron/Malbranchea* (68.2%), *Arachnomyces* (9.1%), *Spiromastigoides* (9.1%), and *Currahmyces* (4.5%), and two newly proposed genera (4.5% each). Based on the results of the phylogenetic study, we synonymized *Auxarthron* with *Malbranchea*, and erected two new genera: *Pseudoarthropsis* and *Pseudomalbranchea*. New species proposed are: *Arachnomyces bostrychodes*, *A. graciliformis*, *Currahmyces sparsispora*, *Malbranchea gymnoascoides*, *M. multiseptata*, *M. stricta*, *Pseudoarthropsis crassispora*, *Pseudomalbranchea gemmata,* and *Spiromastigoides geomycoides*, along with a new combination for *Malbranchea gypsea*. The echinocandins showed the highest in vitro antifungal activity against the studied isolates, followed by terbinafine and posaconazole; in contrast, amphotericin B, fluconazole, itraconazole and 5-fluorocytosine were less active or lacked in vitro activity against these fungi.

**Supplementary Information:**

The online version contains supplementary material available at 10.1186/s43008-021-00075-x.

## INTRODUCTION

The order *Onygenales* includes medically important fungi, such as the dermatophytes and the thermally dimorphic systemic pathogens (*Histoplasm*a, *Coccidioides* and related fungi), which are naturally present in keratinous substrates, in soil, and in freshwater sediments (Currah 1985, 1994; Doveri et al. 2012; Dukik et al. 2017; Hubálek 2000; Hubka et al. 2013; Sharma and Shouche 2019). The genus *Malbranche*a, which is characterized by the production of alternate arthroconidia in branches from the vegetative hyphae, is one of the genus-form of this order; however, it’s pathogenic role in human infections is little known. Only a few cases of fungal infections by species of this genus have been described: *Malbranchea dendritica* has been recovered from lungs, spleen and liver of mice (Sigler and Carmichael 1976), *Malbranchea pulchella* has been suggested as a possible cause of sinusitis (Benda and Corey 1994), and *M. cinnamomea* was recovered from dystrophic nails in patients with underlying chronic illnesses (Lyskova 2007, Salar and Aneja 2007). More recently, *Malbranchea* spp. have been proposed as one of the causative agents of Majocchi’s granuloma (Govind et al. 2017; Durdu et al. 2019). In a study of 245 patients with fungal saprophytic infections of nails and skin, *Malbranchea* spp. were isolated in 1% of skin samples (Lyskova 2007). Other studies demonstrated the coexistence (0.3% of the cases) of *Malbranchea* spp. with the primary pathogen patients with tuberculosis (Benda and Corey 1994; Yahaya et al. 2015).

**Fig. 1:**
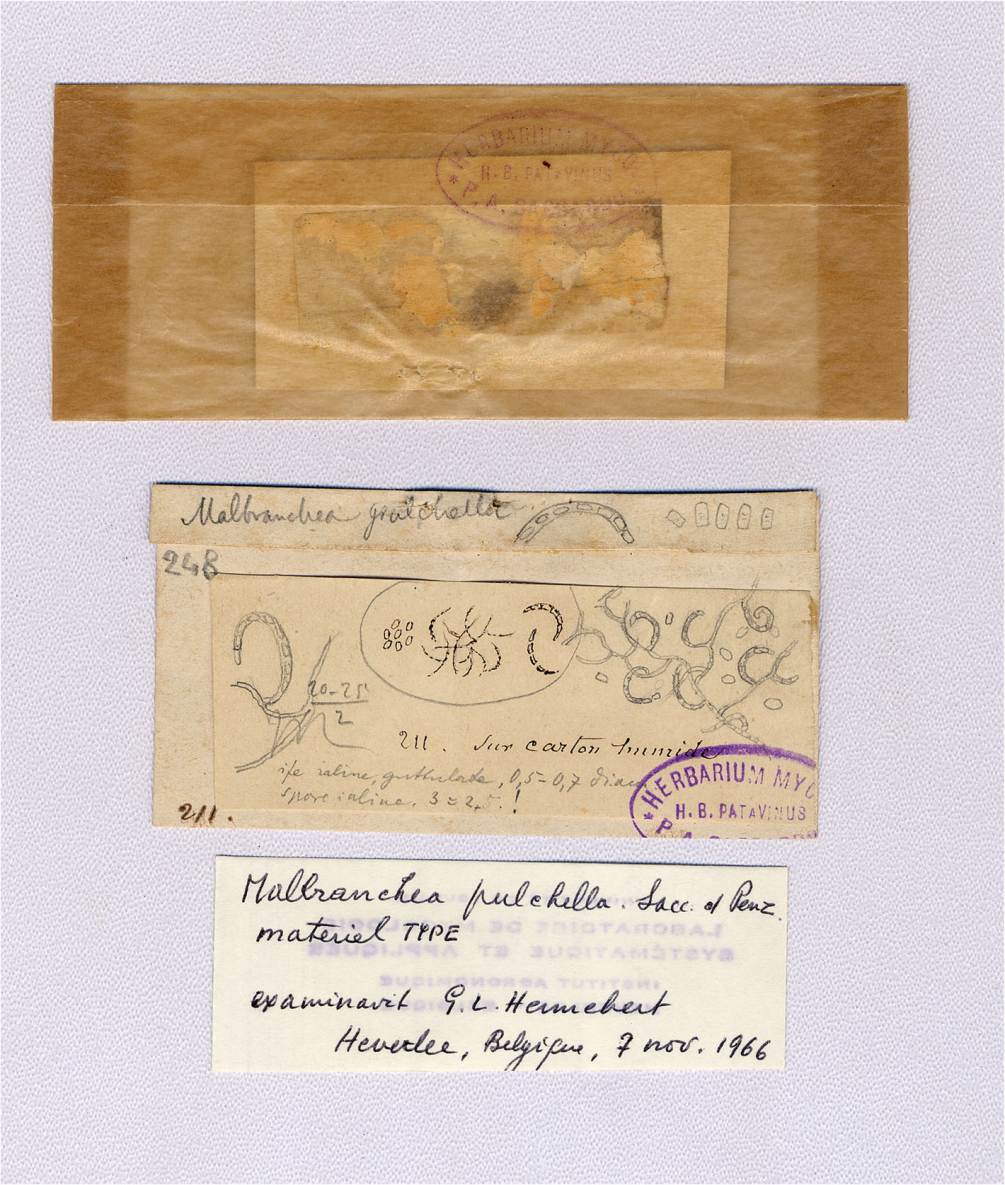
*Malbranchea pulchella* Sacc. & Penzig. Holotype and lectotype. Black ink drawings by A. Malbranche, and pencil drawings by P. A. Saccardo (credits: Rosella Marcucci, erbario micologico di Pier Andrea Saccardo, Università di Padova, Italy)

*Malbranchea* was erected by Saccardo in 1882 for a single species, *Malbranchea pulchella.* It is characterized by alternate arthroconidia originating in curved branches from the vegetative hyphae, which developed on the surface of wet cardboard collected by A. Malbranche in Normandy, France (Fig. 1). Cooney and Emerson reviewed the genus in 1964, providing an appropriated description for mesophilic (*M. pulchella*) and thermophilic (*Malbranchea sulfurea*) species. In a more recent revision by Sigler and Carmichael (1976) 12 species were accepted, while a close relationship with the genus *Auxarthron* (family *Onygenaceae*, order *Onygenales*) was reported, i.e. the species *Auxarthron conjugatum* forms a malbranchea-like asexual morph, and *Malbranchea albolutea* produces a sexual morph related to *Auxarthron*. Also, Sigler and co-workers (2002) connected *Malbranchea filamentosa* with *Auxarthron* based on molecular studies, and also reported the production of fertile ascomata after an in vitro mating of several sexually compatible strains of *M. filamentosa*. The genus *Auxarthro*n produces reddish brown, appendaged gymnothecial ascomata with globose prototunicate 8-spored asci, and globose or oblate, reticulate ascospores (Solé et al. 2002). Some species of this genus, such as *Auxarthron ostraviense* and *A. umbrinum* have been reported as producing onychomycosis in humans (Hubka et al. 2013), and *Auxarthron brunneum*, *A. compactum* and *A. zuffianum* were also isolated from the lungs of kangaroo rats, *A. conjugatum* from lungs of rodents, and *A. umbrinum* from lung of dogs, bats and rodents (Orr et al. 1963; Kuehn et al. 1964).

Malbranchea-like asexual morphs are also present in other taxa of ascomycetes. The genus *Arachnomyces* (family *Arachnomycetaceae*, order Arachnomycetales; Malloch and Cain 1970, Guarro et al. 1993), characterized by the production of brightly coloured cleistothecial ascomata bearing setae, and by the production of an onychocola-like (Sigler et al. 1994) or a malbranchea-like (Udagawa and Uchiyama 1999) asexual morph, have been also implicated in animal and human infections. Specifically, *Arachnomyces nodosetosus* and *Arachnomyces kanei* have been reported as causing nail and skin infections in humans (Sigler and Congly 1990; Sigler et al. 1994; Campbell et al. 1997; Contet-Audonneau et al. 1997; Kane et al. 1997; Koenig et al. 1997; Gupta et al. 1998; Erbagci et al. 2002; Gibas et al. 2002; Llovo et al. 2002; O’Donoghue et al. 2003; Gibas et al. 2004; Stuchlík et al. 2011; Järv 2015; Gupta et al. 2016). More recently, *Arachnomyces peruvianus* has been reported to cause cutaneous infection (Brasch et al. 2017) and *A. glareosus* was isolated from nail and skin samples (Gibas et al. 2004; Sun et al. 2019).

The recently described *Spiromastigoides albida,* isolated from human lung in USA (Stchigel et al. 2017), also produces a malbranchea-like asexual morph. This genus (family *Spiromastigaceae*, *Onygenales*) produces orange gymnothecial ascomata with contorted to coiled appendages and pitted and lenticular ascospores (Kuehn and Orr 1962; Uchiyama et al. 1995; Unterainer et al. 2002; Hirooka et al. 2016).

Due to the limited knowledge of *Malbranchea* and their relatives in human infections, we have studied phenotypically and molecularly a set of malbranchea-like fungal strains from clinical specimens received in a fungal reference centre in the USA. Phylogenetic study and an antifungal susceptibility testing were also carried out.

## MATERIALS AND METHODS

### Fungal strains

Twenty-two malbranchea-like fungal strains (19 from human specimens and three from animals) from different locations in USA were included in this study. The strain number, anatomical source, and geographic origin of the specimens are listed in Table 1. They were provided by the Fungus Testing Laboratory of the University of Texas Health Science Centre at San Antonio (UTHSC; San Antonio, Texas, USA).

**Table 1:** DNA barcodes used to build the phylogenetic tree

Species	Strains^**a**^	GenBank accession #^**b**^		Geographic origin and source
		ITS^**c**^	LSU^**c**^	
*Ajellomyces capsulatus*	UAMH 3536 ^d^	AF038354	AF038354	Alberta, Canada; woman, 25-years-old, biopsy of right middle lobe lung
*Amauroascus niger*	ATCC 22339	MH869547	AY176706	California, U.S.A.; soil
*Amauroascus purpureus*	IFO 32622 ^d^	AJ271564	AY176707	Japan; soil
*Amauroascus volatilis-patellis*	CBS 249.72 ^d^	MH860467	MH872189	Utah, U.S.A.; soil
*Aphanoascus mephitalis*	ATCC 22144	MH859941	AY176725	Ontario, Canada; wolf dung
*Arachniotus verruculosus*	CBS 655.71	NR_145221	AB040684	Utah, U.S.A.; soil
***Arachnomyces bostrychodes*sp. nov*.***	**UTHSCSA DI18-91 = FMR 17685 = CBS 146926**^**d**^	**LR701765**	**LR701766**	**Texas, U.S.A.; human scalp**
*Arachnomyces glareosus*	CBS 116129 ^d^	AY624316	FJ358273	Alberta, Canada; man, 30-years-old, thumb nail
***Arachnomyces graciliformis*sp. nov.**	**UTHSCSA DI18-97 = FMR 17691 = CBS 146927**^**d**^	**LR743667**	**LR743668**	**Massachusetts, U.S.A.; animal bone**
*Arachnomyces gracilis*	UAMH 9756 ^d^	AY123779	–	Uganda; termitarium soil
*Arachnomyces jinanicus*	CGMCC3.14173 ^d^	KY440749	KY440752	Jinan, China; pig farm soil
*Arachnomyces kanei*	UAMH 5908 ^d^	AY123780	–	Toronto, Canada; human nail
*Arachnomyces minimus*	CBS 324.70 ^d^	AY123783	FJ358274	Ontario, Canada; decaying wood
*Arachnomyces nitidus*	UAMH 10536	–	AB075351	Israel; twigs
*Arachnomyces nodosetosus*	CBS 313.90 ^d^	AY123784	AB053452	Saskatchewan, Canada; woman, 67-years-old, onychomycosis
*Arachnomyces peruvianus*	CBS 112.54 ^d^	MF572315	MH868792	Peru; *Globodera rostochiensis* cyst
*Arachnomyces pilosus*	CBS 250.93 ^d^	MF572320	MF572325	Catalonia, Spain; river sediment
*Arachnomyces scleroticus*	UAMH 7183 ^d^	AY123785	–	Sulawesi, Indonesia; poultry farm soil
*Arthroderma curreyi*	CBS 353.66 ^d^	MH858822	MH870459	UK; unknown
*Arthroderma onychocola*	CBS 132920 ^d^	KT155794	KT155124	Prague, Czech Republic; human nail
*Ascosphaera apis*	CBS 252.32	–	AY004344	København, Denmark; *A. mellifera*
*Ascosphaera subglobosa*	A.A. Wynns 5004 (C) ^d^	NR_137060	HQ540517	Utah, U.S.A.; pollen provisions of *M. rotundata*
*Auxarthronopsis bandhavgarhensis*	NFCCI 2185 ^d^	HQ164436	NG_057012	Bandhavgarh, India; soil
*Auxarthronopsis guizhouensis*	CGMCC3.17910 ^d^	KU746668	KU746714	Guizhou, China; air
*Blastomyces percusus*	CBS 139878 ^d^	NR_153647	KY195971	Israel; human granulomatous lesions
*Canomyces reticulatus*	MCC 1486 ^d^	MK340501	MK340502	Maharashtra, India; soil
*Chrysosporium keratinophilum*	CBS 392.67	MH859002	AY176730	New Zealand; soil
*Chrysosporium tropicum*	MUCL 10068 ^d^	MH858134	AY176731	Guadalcanal, Solomon islands; woollen overcoat
*Currahmyces indicus*	MCC 1548 ^d^	MK340498	MK340499	Maharashtra, India; hen resting area
***Currahmyces sparsispora*sp. nov.**	**UTHSCSA DI18-89 = FMR 17683 = CBS 146929**^**d**^	**LR723272**	**LR723273**	**Florida, U.S.A.; human sputum**
*Gymnoascus reesii*	CBS 410.72	MH860507	MH872224	California, U.S.A.; soil
*Helicoarthrosporum mellicola*	CBS 143838 ^d^	LR761645	LT906535	Granada, Spain; honey
*Helicoarthrosporum mellicola*	FMR 15673	LR761646	LT978462	Valencia, Spain; honey
***Malbranchea albolutea***	**UTHSCSA DI18-85 = FMR 17679**	**LR701834**	**LR701835**	**Texas, U.S.A.; human BAL**
***Malbranchea albolutea***	**UTHSCSA DI18-95 = FMR 17689**	**LR701836**	**LR701837**	**Texas, U.S.A.; human BAL**
*Malbranchea albolutea*	CBS 125.77 ^d^	MH861039	MH872808	Utah, U.S.A.; soil
***Malbranchea aurantiaca***	**UTHSCSA DI18-94 = FMR 17688**	**LR701824**	**LR701825**	**California, U.S.A.; animal**
***Malbranchea aurantiaca***	**UTHSCSA DI18-88 = FMR 17682**	**LR701826**	**LR701827**	**Texas, U.S.A.; animal skin lesion**
*Malbranchea aurantiaca*	CBS 127.77 ^d^	NR_157447	AB040704	Utah, U.S.A.; culture contaminant
*Malbranchea californiensis*	ATCC 15600 ^d^	MH858121	NG_056947	California, U.S.A.; dung of pack rat
*Malbranchea chinense*	CGMCC3.19572	MK329076	MK328981	Guangxi, Luotian Cave, China; Soil
*Malbranchea chrysosporioidea*	CBS 128.77 ^d^	AB361632	AB359413	Arizona, U.S.A.; soil
*Malbranchea circinata*	ATCC 34526 ^d^	MN627784	MN627782	Utah, U.S.A.; soil
***Malbranchea conjugata***	**UTHSCSA DI18-105 = FMR 17699**	**LR701828**	**LR701829**	**Florida, U.S.A.; human lung tissue**
***Malbranchea conjugata***	**UTHSCSA DI18-103 = FMR 17697**	**LR701830**	**LR701831**	**Texas, U.S.A.; human BAL**
*Malbranchea conjugata*	CBS 247.58	NR_121475	HF545313	Arizona, U.S.A.; soil
*Malbranchea dendritica*	CBS 131.77 ^d^	AY177310	AB359416	Utah, U.S.A.; soil
*Malbranchea filamentosa*	CBS 581.82 ^d^	NR_111136	AB359417	Argentina; soil
*Malbranchea flava*	CBS 132.77 ^d^	AB361633	AB359418	California, U.S.A.; soil
*Malbranchea flavorosea*	ATCC 34529 ^d^	NR 158362	AB359419	California, U.S.A.; soil
***Malbranchea flocciformis***	**UTHSCSA DI18-104 = FMR 17698**	**LR701822**	**LR701823**	**Texas, U.S.A.; human skin**
*Malbranchea flocciformis*	CBS 133.77 ^d^	AB361634	AB359420	France; saline soil
*Malbranchea fulva*	CBS 135.77 ^d^	NR_157444	AB359422	Utah, U.S.A.; air
***Malbranchea gymnoascoides*sp. nov.**	**UTHSCSA DI18-87 = FMR 17681 = CBS 146930**^**d**^	**LR701757**	**LR701758**	**Texas, U.S.A.; human BAL**
*Malbranchea guangxiense*	CGMCC3.19634	MK329080	MK328985	Guangxi, E’gu Cave, China; Soil
*Malbranchea kuehnii*	CBS 539.72 ^d^	NR_103573	NG_056928	Unkown; dung
*Malbranchea longispora*	FMR 12768 ^d^	HG326873	HG326874	Beija, Portugal; soil
***Malbranchea multiseptata*sp. nov.**	**UTHSCSA DI18-101 = FMR 17695 = CBS 146931**^**d**^	**LR701759**	**LR701760**	**Texas, U.S.A.; human BAL**
*Malbranchea ostraviense*	CBS 132919 ^d^	NR_121474	–	Ostrava, Czech Republic; fingernail sample
*Malbranchea pseudauxarthron*	IFO 31701 = CBS 657.71 = ATCC 22158 = NRRL 5132	MH860293	KY014424	Utah, U.S.A.; domestic rabbit dung
*Malbranchea pulchella*	CBS 202.38	AB361638	AB359426	Italy; unknown
***Malbranchea stricta*sp. nov.**	**UTHSCSA DI18-86 = FMR 17680 = CBS 146932**^**d**^	**LR701638**	**LR701639**	**Florida, U.S.A.; human nail**
*Malbranchea* sp.^e^	CBS 319.61	MH858065	MH869635	California, U.S.A.; soil
***Malbranchea umbrina***	**UTHSCSA DI18-106 = FMR 17700**	**LR701814**	**LR701815**	**Colorado, U.S.A.; human BAL**
***Malbranchea umbrina***	**UTHSCSA DI18-107 = FMR 17701**	**LR701816**	**LR701817**	**Colorado, U.S.A.; human sinus**
***Malbranchea umbrina***	**UTHSCSA DI18-100 = FMR 17694**	**LR701818**	**LR701819**	**Baltimore, U.S.A.; human wound**
***Malbranchea umbrina***	**UTHSCSA DI18-99 = FMR 17693**	**LR701820**	**LR701821**	**Washington DC, U.S.A.; human nail**
*Malbranchea umbrina*	CBS 105.09 ^d^	MH854591	MH866116	UK; soil
*Malbranchea umbrina*	CBS 226.58	MH857765	MH869296	Unknown
*Malbranchea umbrina*	CBS 261.52	MH857026	MH868556	UK; soil
***Malbranchea zuffiana***	**UTHSCSA DI18-96 = FMR 17690**	**LR701832**	**LR701833**	**Washington DC, U.S.A.; human wound**
*Malbranchea zuffiana*	CBS 219.58 ^d^	MH869293	AY176712	Texas, U.S.A.; prairie dog lung
*Nannizziopsis guarroi*	CBS 124553 ^d^	MH863384	MH874904	Barcelona, Spain; iguana skin
*Nannizziopsis vriesii*	ATCC 22444 ^d^	AJ131687	AY176715	The Netherlands; Ameiva (lizard) skin and lung
*Neogymnomyces demonbreunii*	CBS 427.70	AJ315842	AY176716	Missouri, U.S.A.; unknown
*Onychocola canadensis*	CBS 109438	–	KT154998	Italy; nail and skin scrapings
*Paracoccidioides brasiliensis*	UAMH 8037 ^d^	AF038360	AF038360	Alberta, Canada; man, 59-years-old, lung biopsy
*Pseudoarthropsis cirrhata*	CBS 628.83 ^d^	–	NG_060792	Schiphol, The Netherlands; wall sample
***Pseudoarthropsis crassispora*sp. nov.**	**UTHSCSA DI18-98 = FMR 17692 = CBS 146928**^**d**^	**LR701763**	**LR701764**	**Minnesota, U.S.A.; human BAL**
***Pseudomalbranchea gemmata*gen. nov. et sp. nov.**	**UTHSCSA DI18-90 = FMR 17684 = CBS 146933**^**d**^	**LR701761**	**LR701762**	**Florida, U.S.A.; human BAL**
*Pseudospiromastix tentaculata*	CBS 184.9210536	AY527406	LN867603	Hiram, Somalia; soil
*Renispora flavissima*	CBS 708.79 ^d^	AF299348	AY176719	Kansas, U.S.A.; soil in barn housing *M. velifer*
*Spiromastigoides alatosporus*	CBS 457.73 ^d^	MH860740	AB075342	Madras, India; *V. sinensis* rhizosphere
*Spiromastigoides albina*	CBS 139510 ^d^	LN867606	LN867602	Texas, U.S.A.; human lung biopsy
*Spiromastigoides asexualis*	CBS 136728 ^d^	KJ880032	LN867603	Phoenix, U.S.A.; discospondylitis material from a German shepherd dog
*Spiromastigoides curvata*	JCM 11275 ^d^	KP119631	KP119644	México; contaminant of a strain of *Histoplasma capsulatum*
*Spiromastigoides frutex*	CBS 138266 ^d^	KP119632	KP119645	Nayarit, Mexico; house dust, rental studio
***Spiromastigoides geomycoides*sp. nov.**	**UTHSCSA DI18-92 = FMR 17686**	**LR701769**	**LR701770**	**Minnesota, U.S.A.; human blood**
***Spiromastigoides geomycoides*sp. nov.**	**UTHSCSA DI18-102 = FMR 17696 = CBS 146934**^**d**^	**LR701767**	**LR701768**	**Illinois, U.S.A.; human skin foot**
*Spiromastigoides gypsea*	CBS 134.77 ^d^	KT155798	NG_063935	California, U.S.A.; soil
*Spiromastigoides kosraensis*	CBS 138267 ^d^	KP119633	KP119646	Kosrae, Micronesia; house dust
*Spiromastigoides pyramidalis*	CBS 138269 ^d^	KP119636	KP119649	Australia; house dust
*Spiromastigoides sugiyamae*	JCM 11276 ^d^	LN867608	AB040680	Japan; soil
*Spiromastigoides warcupii*	CBS 576.63 ^d^	LN867609	AB040679	Australia; soil
*Strongyloarthrosporum capsulatus*	CBS 143841 ^d^	LR760230	LT906534	Toledo, Spain; honey
*Trichophyton bullosum*	CBS 363.35 ^d^	NR_144895	NG_058191	Unkown
*Uncinocarpus reesii*	ATCC 34533	MH861035	AY176724	Australia; feather

^a^*ATCC* American Type Culture Collection, Virginia, USA, *BCCM/MUCL* Mycothèque de l’Université Catholique de Louvain, Louvain-la-Neuve, Belgium, *CBS* Culture collection of the Westerdijk Biodiversity Institute, Utrech, The Netherlands, *CGMCC* China General Microbiological Culture Collection Center, Beijing, China, *FMR* Facultat de Medicina, Reus, Spain, *IFO* Institute for Fermentation Culture Collection, Osaka, Japan, *JCM* Japan Collection of Microorganisms, Tsukuba, Japan, *MCC* Microbial Culture Collection, Universite of Pune Campus Ganeshkhind, India, *NFCCI* National Fungal Culture Collection of India, Maharastra, India, *UAMH* University of Alberta Microfungus Collection and Herbarium, Alberta, Canada, *UTHSC* Fungus Testing Laboratory, University of Texas Health Science Center at San Antonio, San Antonio, Texas, United States

^b^Strains studied by us are indicated in bold

^c^*ITS* internal transcribed spacer region 1 and 2 including 5.8S nrDNA, *LSU* large subunit of the nrRNA gene

^d^Ex-type strain

^e^Strain formerly assigned to *Auxarthron thaxteri* (a species synonymized with *Malbranchea umbrina*)

### Phenotypic study

For cultural characterization, suspensions of conidia were prepared in a semi-solid medium (0.2% agar; 0.05% Tween 80) and inoculated onto phytone yeast extract agar (PYE; Becton, Dickinson & Company, Sparks, MD, USA; Carmichael and Kraus 1959), potato dextrose agar (PDA; Pronadisa, Madrid, Spain; Hawksworth et al. 1995), oatmeal agar (OA; 30 g of filtered oat flakes, 15 g agar-agar, 1 L tap water; Samson et al. 2010), bromocresol purple-milk solids-glucose agar (BCP-MS-G; 80 g skim milk powder, 40 g glucose, 10 mL of 1.6% of bromocresol purple in 95% ethanol, 30 g agar-agar,1 L tap water; Kane and Smitka 1978), and test opacity tween medium (TOTM; 10 g bacteriological peptone, 5 g NaCl, 1 g CaCl_2_, 5 mL Tween, 5 mL Tween 80, 15 g agar-agar, 1 L tap water; Slifkin 2000). Colonies were characterized after 14 days at 25 °C in the dark. Potato dextrose agar (PDA) was used to determine the cardinal temperatures of growth. Colour notations were taken according to Kornerup and Wanscher (1978). Christensen’s urea agar (EMD Millipore, Darmstadt, Germany; Christensen 1946) was inoculated and incubated for 4 days at 25 °C in the dark to detect the production of urease. Cycloheximide tolerance was tested growing the fungal strains on Sabouraud dextrose agar (SDA; Pronadisa, Spain) supplemented with 0.2% cycloheximide (Sigma, USA) at 30 °C for two wk. Fungal tolerance to NaCl was evaluated on SDA adding 3, 10 and 20% w/w NaCl, with the same incubation conditions as previously described. The microscopic structures were characterized and measured from wet mountings of slide cultures, using water and 60% lactic acid. Photo micrographs were taken using a Zeiss Axio-Imager M1 light microscope (Oberkochen, Germany) with a DeltaPix Infinity X digital camera using Nomarski differential interference contrast. The descriptions of the taxonomical novelties were submitted to MycoBank (https://www.mycobank.org/; Crous et al. 2004).

### DNA extraction, amplification and sequencing

Total DNA was extracted as previously described (Valenzuela-Lopez et al. 2018), and the following phylogenetic markers were amplified: the internal transcribed spacers (ITS) (ITS5/ITS4 primers; White et al. 1990, and a fragment of the large subunit (LSU) gene (LR0R/LR5 primers; Vilgalys and Hester 1990; Rehner and Samuels 1994) of the nrDNA. Amplicons were sequenced at Macrogen Europe (Macrogen Inc., Madrid, Spain) using the same pair of primers. Consensus sequences were obtained by SeqMan software v. 7 (DNAStar Lasergene, Madison, WI, USA). Sequences generated in this work were deposited in GenBank (Table 1).

### Phylogenetic analysis

A preliminary molecular identification of the isolates was carried out with ITS and LSU nucleotide sequences using BLAST (https://blast.ncbi.nlm.nih.gov/Blast.cgi), and only the sequences of ex-type or reference strains from GenBank were included for identification. A maximum level of identity (MLI) ≥ 98% was used for species-level and < 98% for genus-level identification. A maximum-likelihood (ML) and Bayesian-inference (BI) phylogenetic analyses of the concatenated ITS-LSU sequences were performed in order to determine the phylogenetic placement of our clinical strains. Species of the order *Arachnomycetales* were used as outgroup. The sequence alignments and ML / BI analyses were performed according to Valenzuela-Lopez et al. (2018). The final matrices used for the phylogenetic analysis were deposited in TreeBASE (www.treebase.org; accession number: 25068).

### Antifungal susceptibility testing

In vitro antifungal susceptibility testing was carried out following the broth microdilution method from the Clinical and Laboratory Standards Institute (CLSI) protocol M38 (CLSI 2017) with some modifications. The antifungal drugs tested were amphotericin B (AMB), fluconazole (FLC), voriconazole (VRC), itraconazole (ITC), posaconazole (PSC), anidulafungin (AFG), caspofungin (CFG), micafungin (MFG), terbinafine (TRB), and 5-fluorocytosine (5-FC). Briefly, incubation media, temperature and time were set to the sporulation requirements of every strain, and conidia suspensions were inoculated into the microdilution trays after being adjusted by haemocytometer counts. Incubation was set at 35 °C (without light or agitation) until the drug-free well displayed a visible fungal growth (minimum 48 h; maximum 10 days) for quantification of the Minimal Effective Concentrations (MEC) for the echinocandins and the Minimal Inhibitory Concentrations (MIC) for the other tested antifungals. The MEC value was stablished as the lowest drug concentration at which short, stubby and highly branched hyphae were observed, while the MIC value was defined as the lowest concentration that completely inhibited the fungal growth. *C. parapsilosis* ATCC 22019 was used as the quality control strain in all experiments.

## RESULTS

### Fungal diversity

Table 1 shows the identity of the 22 fungal strains studied. The highest number of strains corresponded to *Auxarthron umbrinum* (4), followed by *A. alboluteum* (2), *A. conjugatum* (2), and *Malbranchea aurantiaca* (2). *Auxarthron zuffianum*, *Currahmyces indicus* and *M. flocciformis* were represented by one strain each. Eight strains were only identified at genus-level (three belonging to *Malbranchea*, two to *Spiromastigoides*, two to *Arachnomyces*, and one to *Arthropsis*), one strain (FMR 17684) only at family-level (*Onygenaceae*).

### Molecular phylogeny

**Fig. 2:**
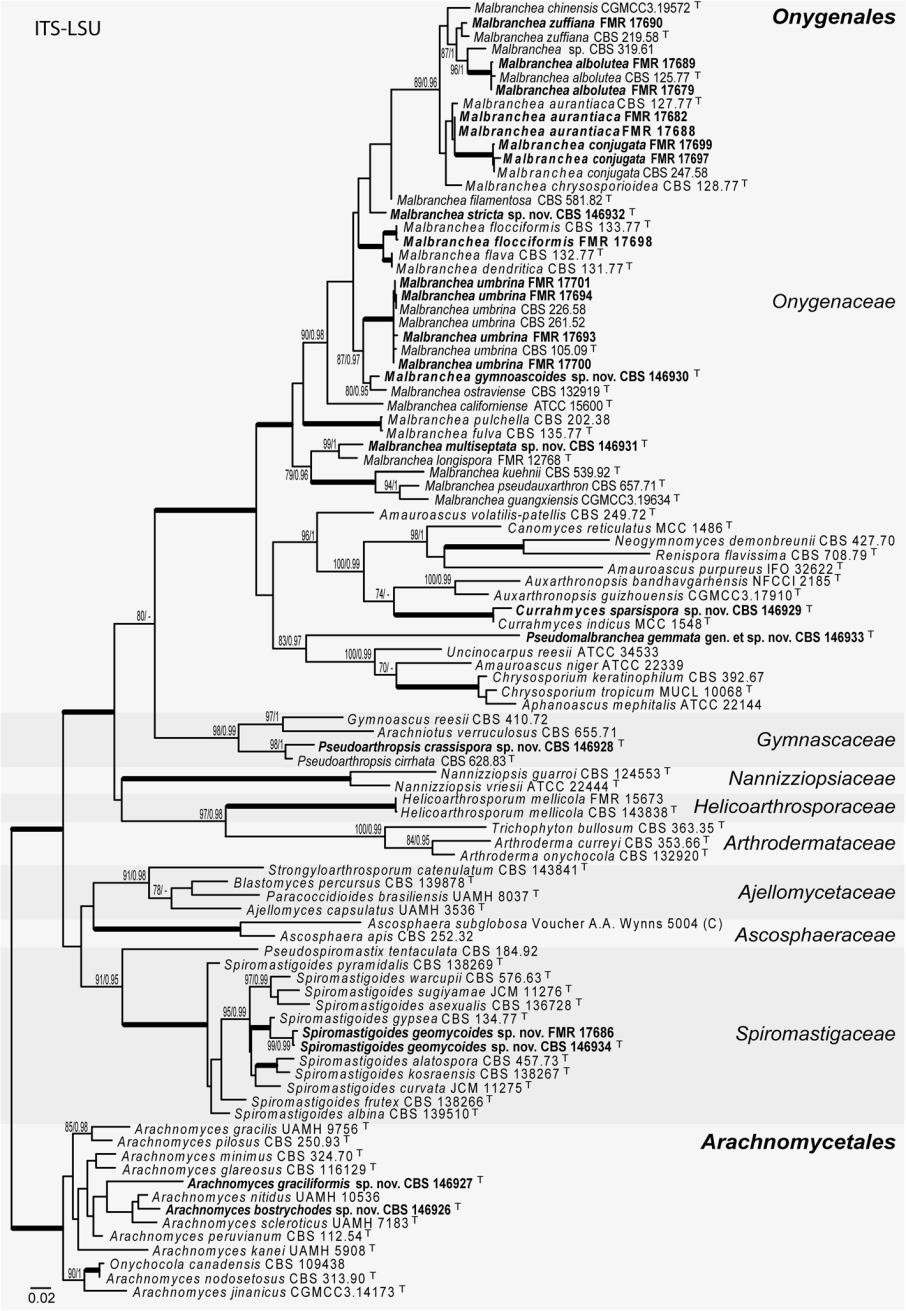
ML phylogenetic tree based on the analysis of ITS-LSU nucleotide sequences for the 22 clinical fungi from the USA. Bootstrap support values/Bayesian posterior probability scores of 70/0.95 and higher are indicated on the nodes. ^T^ = ex type. Fully supported branched (100% BS /1 PP) are indicated in bold. Strains identified by us are in bold. *Arachnomyces* spp. were chosen as out-group. The sequences used in this analysis are in Table 1

Our phylogenetic study included 92 sequences corresponding to 75 species with a total of 1213 characters (700 ITS and 513 LSU) including gaps, of which 579 were parsimony informative (402 ITS and 177 LSU). The ML analysis was congruent with that obtained in the BI analysis, both displaying trees with similar topologies. The datasets did not show conflict with the tree topologies for the 70% reciprocal bootstrap trees, which allowed the two genes to be combined for the multi-locus analysis (Fig. 2). (single gene-based phylogenies are as supplemental material Figures S1 and S2).

Twenty of our strains were placed into a main clade corresponding to the members of the *Onygenales* (100% BS / 1 PP), while two were placed in the *Arachnomycetales* (100% BS / 1 PP) (Fig. 2). The *Onygenales* clade was divided into eight clades corresponding to the families *Onygenaceae* (100% BS / 1 PP), *Gymnascaceae* (98% BS / 0.99 PP), *Nannizziopsiaceae* (100% BS / 1 PP), *Helicoarthrosporaceae* (100% BS / 1 PP), *Arthrodermataceae* (100% BS / 0.99 PP), *Ajellomycetaceae* (91% BS / 0.98 PP), *Ascosphaeraceae* (100% BS / 1 PP), and *Spiromastigaceae* (91% BS / 0.95 PP), which included a basal terminal branch for *Pseudospiromastix tentaculata*. Most of our strains (17/22) were distributed into several subclades of the *Onygenaceae*: 15/22 into *Auxarthron*/*Malbranchea* subclade (100% BS / 1 PP), one into a terminal branch (FMR 17683) together *Currahmyces indicus* (100% BS / 1 PP), and another one (FMR 17684) into a distant, independent terminal branch. One strain (FMR 17692) was placed into the *Gymnascaceae*, in a terminal branch together with *Arthropsis cirrhata* (98% BS / 1 PP). The *Spiromastigaceae* included the last two strains (FMR 17686 and FMR 17696 (CBS 146934)), placed into a terminal branch together *Malbranchea gypsea* (100% BS / 1 PP).

### TAXONOMY

***Arachnomyces***

Since the strains FMR 17685 and FMR 17691 represented two species of *Arachnomyces* that were different from the other species of the genus*,* they are described as new, here.

***Arachnomyces bostrychodes*** Rodr.-Andr., Cano & Stchigel, **sp. nov*.***

**(**Fig. 3)

**Fig. 3:**
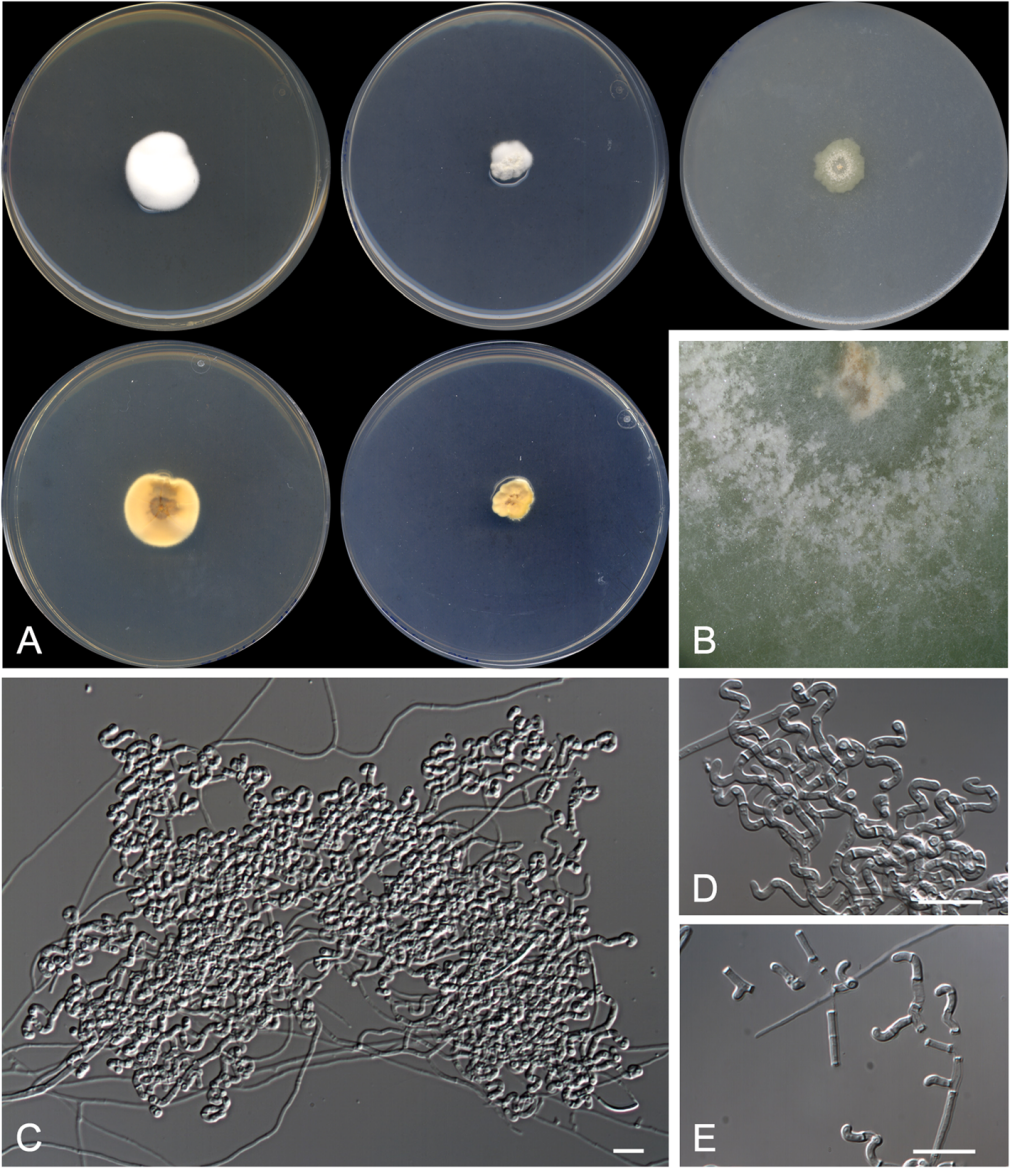
*Arachnomyces bostrychodes* CBS 146926 ^T^. **a** Colonies on PYE, PDA and OA after 14 d at 25 °C, from left to right (top row, surface; bottom row, reverse). **b** Detail of the colony on OA. **c**, **d** Sinuous, contorted to coiled fertile hyphae. **e** Arthroconidia. Scale bar = 10 μm

MycoBank MB 834921

*Etymology:* From Greek *βοστρυχος*-, curl, due to the appearance of the reproductive hyphae.

*Diagnosis*: The phylogenetically closest species to *Arachnomyces bostrychodes* is *A. peruvianum* (Fig. 2). Nevertheless, *A. botrychodes* lacks a sexual morph and racket hyphae (both present in *A. peruvianum*), and produces longer conidia than *A. peruvianum* (4.0–8.0 × 1.0–2.0 μm vs. 4.0–5.0 × 1.0–3.0 μm); also, *A. bostrychodes* grows more slowly on OA (13–14 mm diam after 2 wk. at 25 °C) than *A. peruvianum* (30 mm diam) (Cain 1957; Brasch et al. 2017). *Arachnomyces bostrychodes* morphologically resembles *Arachnomyces gracilis*, but the former grows faster, produces more strongly contorted branches and lacks of a sexual morph.

*Type*: **USA**: *Texas*: from a human scalp, 2008, *N. Wiederhold* (CBS H-24452 – holotype; CBS 146926 = FMR 17685 = UTHSCSA DI18-91 – ex-type cultures; LSU/ITS sequences GenBank LR701766/LR701765).

*Description: Vegetative hyphae* hyaline, septate, branched, smooth- and thin-walled, 1.0–2.0 μm wide*. Fertile hyphae* well-differentiated, arising as lateral branches from the vegetative hyphae, successively branching to form dense clusters, arcuate, sinuous, contorted or tightly curled, 1.0–2.0 μm wide, forming randomly intercalary and terminally arthroconidia. *Conidia* enteroarthric, hyaline, one-celled, smooth-walled, cylindrical, barrel-shaped, and finger-like-shaped when terminal, 4.0–8.0 × 1.0–2.0 μm, mostly curved and truncated at one or (mostly) both ends, separated from the fertile hyphae by rhexolysis. *Chlamydospores, racquet hyphae*, *setae*, and s*exual morph* not observed.

*Culture characteristics*: Colonies on PYE reaching 19–20 mm diam after 2 wk. at 25 °C, elevated, cottony, margins regular, white (5A1), sporulation absent; reverse light orange (5A4). Colonies on PDA reaching 11–12 mm diam after 2 wk. at 25 °C, elevated, velvety with floccose patches, margins regular, yellowish white (4A2), sporulation abundant; reverse greyish yellow (4B6). Colonies on PDA reaching 13–14 mm diam after 2 wk. at 30 °C, slightly elevated, velvety to floccose, regular margins, white (4A1), sporulation sparse; reverse, greyish yellow (4B6). Colonies on OA researching 13–14 mm diam after 2 wk. at 25 °C, flattened, smooth and granulose, irregular margins, yellowish white (2A2) at centre and light yellow (2A5) at edge, sporulation abundant. Exudate and diffusible pigment absent.

Minimum, optimal and maximum temperature of growth (on PDA): 10 °C, 30 °C, and 37 °C, respectively. Non-haemolytic. Casein not hydrolysed. Not inhibited by cycloheximide. Urease and esterase (TOTM) tests positive. Growth occurs at NaCl 10% w/w, but not at 20% w/w.

***Arachnomyces graciliformis*** Rodr.-Andr., Stchigel & Cano, **sp. nov.**

(Fig. 4)

**Fig. 4:**
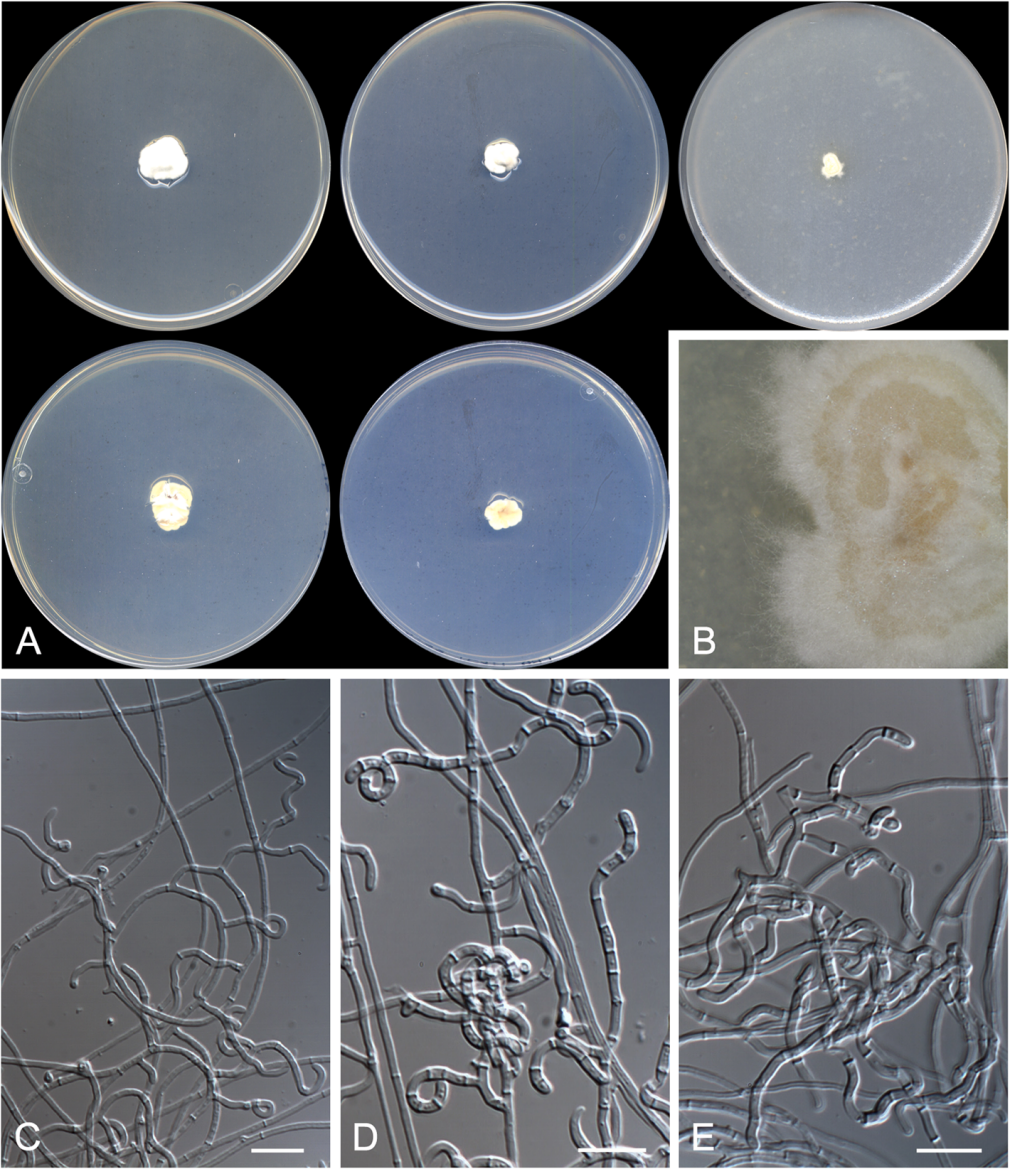
*Arachnomyces graciliformis* CBS 146927 ^T^. **a** Colonies on PYE, PDA and OA after 14 d at 25 °C, from left to right (top row, surface; bottom row, reverse). **b** Detail of the colony on OA. **c**–**e** Contorted, apically coiled fertile hyphae bearing arthroconidia. Scale bar = 10 μm

MycoBank MB 834923

*Etymology:* Recalling the morphological similarity with *Arachnomyces gracilis*.

*Diagnosis*: *Arachnomyces graciliformis* is phylogenetically close to *A. glareosus* and to *A. minimus* (Fig. 2). These three species form a clade together with *A. nodosetosus* and *A. jinanicus* (84 BS / 1 PP). Unlike *A. glareosus* and *A. minimus*, *A. graciliformis* does not produces racquet hyphae nor sexual morph (Gibas et al. 2004) but produces longer conidia than *A. glareosus* (4.0–10.0 × 1.5–2.0 μm vs. 2.5–4.5 × 1.5–2.0 μm), which are not produced by *A. minimus*. *Arachnomyces graciliformis* morphologically resembles *A. gracilis*, but the former grows more slowly, produces more twisted fertile branches and does not form a sexual morph (Udagawa and Uchiyama 1999).

*Type*: **USA**: *Massachusetts*: from an animal’s bone, 2012, *N. Wiederhold* (CBS H-24453 – holotype; CBS 146927 = FMR 17691 = UTHSCSA DI18-97 – ex-type cultures; LSU/ITS sequences GenBank LR743668/LR743667).

*Description: Vegetative hyphae* hyaline, septate, branched, smooth- and thin-walled, 1.0–2.0 μm wide. *Fertile hyphae* well-differentiated, arising as lateral branches from the vegetative hyphae, branching repeatedly, sinuous to arcuate or apically coiled, 1.5–2.0 μm wide, forming randomly intercalary and terminally arthroconidia. *Conidia* enteroarthric, hyaline, unicellular, smooth- and thin-walled, cylindrical or finger-like-shaped when terminal, 4.0–10.0 × 1.5–2.0 μm, mostly curved, detached from the fertile hyphae by rhexolysis. *Chlamydospores*, *racquet hyphae*, *setae*, and *sexual morph* not observed.

*Culture characteristics*: Colonies on PYE reaching 12–13 mm diam after 2 wk. at 25 °C, elevated, velvety to floccose, margins regular, slightly furrowed, yellowish white (3A2), sporulation absent; reverse greyish orange (5B3). Colonies on PDA reaching 9–10 mm diam after 2 wk. at 25 °C, slightly elevated, velvety to floccose, margins regular, slightly furrowed, yellowish white (1A2), sporulation absent; reverse greyish yellow (4B3). Colonies on PDA reaching 3–4 mm diam after 2 wk. at 30 °C, slightly elevated, velvety to floccose, margins regular, slightly furrowed, yellowish white (1A2), sporulation absent; reverse, greyish yellow (4B3). Colonies on OA researching 6–7 mm diam after 2 wk. at 25 °C, flattened, velvety and granulose, margins irregular, pale yellow (4A3), sporulation absent (conidia appear after 5–6 wk. incubation). Exudate and diffusible pigment absent. Minimum, optimal and maximum temperature of growth (on PDA): 10 °C, 25 °C, and 30 °C, respectively. Non-haemolytic. Casein not hydrolysed. Not inhibited by cycloheximide. Urease and esterase tests positive. Growth occurs at NaCl 10% w/w, but not at 20% w/w.

***Currahmyces***

Due to the strain FMR 17683 being placed into a terminal branch of *Onygenaceae* together with *Currahmyces indicus* (Sharma and Shouche 2019), and because they differ molecularly and phenotypically, we erect the new species *Currahmyces sparsispora*.

***Currahmyces sparsispora*** Rodr.-Andr., Cano & Stchigel, **sp. nov.**

**(**Fig. 5)

**Fig. 5:**
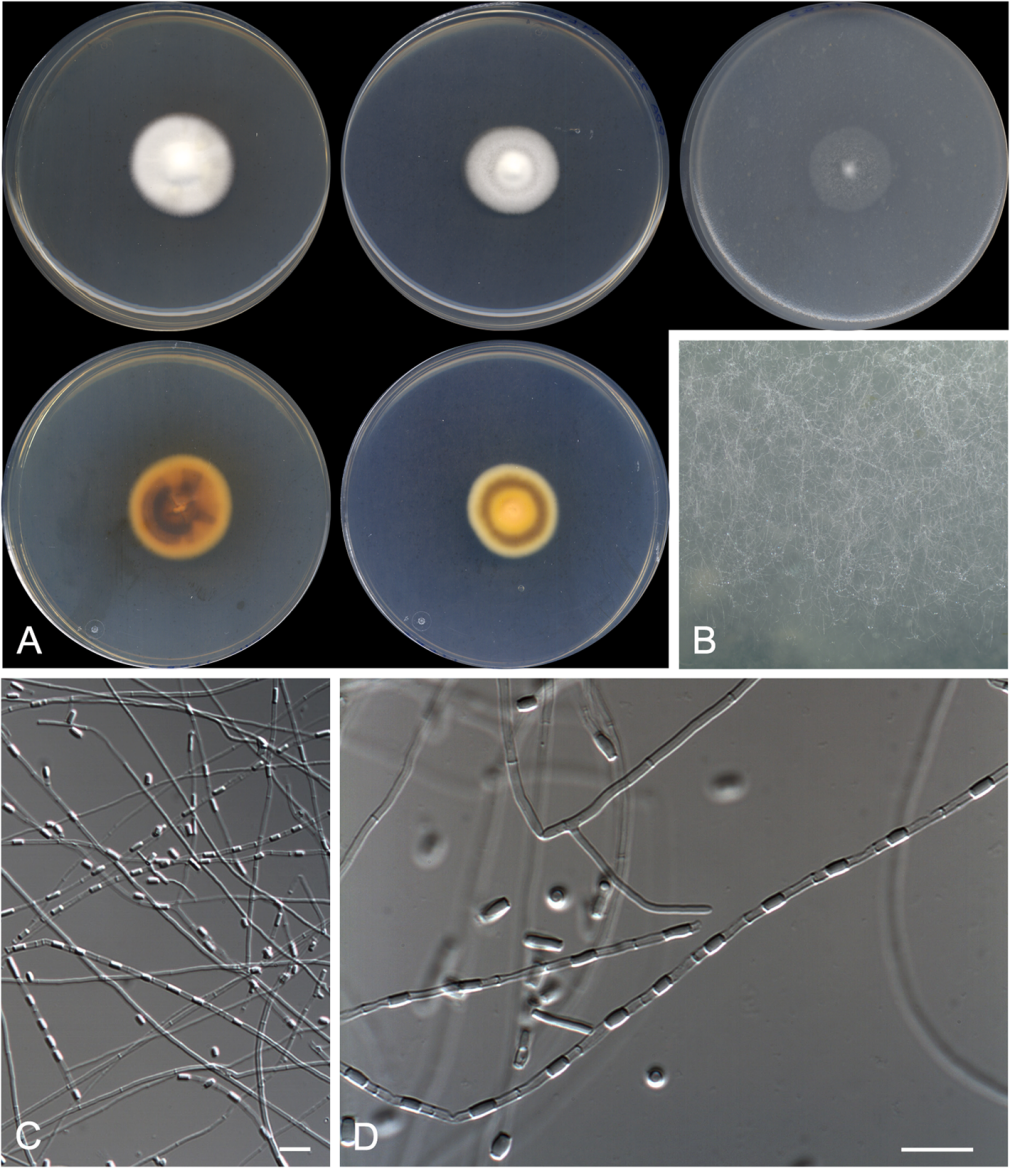
*Currahmyces sparsispora* CBS 146929 ^T^. **a** Colonies on PYE, PDA and OA after 14 d at 25 °C, from left to right (top row, surface; bottom row, reverse). **b** Detail of the colony on OA. **c**–**d** Intercalary arthroconidia along the fertile hyphae. Scale bar = 10 μm

MycoBank MB 835692

*Etymology:* From Latin *sparsa-*, splashed, −*sporarum*, spore, due to the disposition of the conidia along the hyphae.

*Diagnosis*: *Currahmyces sparsispora* is phylogenetically close to *C. indicus*; however, they can be differentiated because the former has broader hyphae (1.5–2.0 μm vs. 0.7–1.1 μm) and lacks a sexual morph (typical gymnothecial ascomata are produced on hair-baited soil plates by *C. indicus*).

*Type*: **USA**: *Florida*: from human sputum, 2007, *N. Wiederhold* (CBS H-24455 – holotype; CBS 146929 = FMR 17683 = UTHSCSA DI18-89 – ex-type cultures; LSU/ITS sequences GenBank LR723273/LR723273).

*Description: Vegetative hyphae* septate, hyaline, smooth- and thin-walled, mostly straight, rarely branched, 1.5–2.0 μm wide. *Fertile hyphae* undifferentiated from the vegetative hyphae. *Conidia* enteroarthric, hyaline, unicellular, smooth- and thin-walled, disposed relatively far from each other along the fertile hyphae, separated by 1–2 evanescent connective cells, cylindrical to slightly barrel-shaped, 3.0–12.0 × 1.0–2.0 μm, separated by rhexolysis. *Chlamydospores*, *racquet hyphae*, *setae*, and *sexual morph* not observed.

*Culture characteristics*: Colonies on PYE reaching 27–28 mm diam after 2 wk. at 25 °C, slightly elevated, velvety to floccose, margins regular, pale orange (5A3) at centre and white (5A1) at edge, sporulation sparse; reverse orange (5A6). Colonies on PDA reaching 23–24 mm diam after 2 wk. at 25 °C, slightly elevated, velvety, margins regular, light orange (5A5) at centre and orange white (5A2) at edge, sporulation sparse; reverse deep orange (6A8). Colonies on PDA reaching 30–31 mm diam after 2 wk. at 30 °C, slightly elevated, velvety, slightly furrowed, margins regular, orange (5A6), sporulation sparse; reverse brownish orange (6C8). Colonies on OA reaching 20–21 mm diam after 2 wk. at 25 °C, slightly elevated, velvety, margins regular, orange white (5A2) at centre and white (5A1) at edge, sporulation sparse. Exudate and diffusible pigment absent in all culture media tested. Minimum, optimal and maximum temperature of growth on PDA: 10 °C, 30 °C, and 37 °C, respectively. Haemolytic. Casein not hydrolysed. Not inhibited by cycloheximide. Urease and esterase tests positive. Growth occurs at NaCl 3% w/w and 10% w/w, but not at 20% w/w.

***Malbranchea***

An emended description of the genus *Malbranchea* is provided as follows:

***Malbranchea*** Sacc., *Michelia* **2**(no. 8): 639 (1882).

MycoBank MB 8833.

*Description: Vegetative hyphae* septate, hyaline, smooth- and thin-walled, straight or branched. *Asexual morph* consisting in undifferentiated fertile hyphae, and/or well-differentiated lateral branches, curved or not, which form randomly or basipetally terminal and intercalary arthroconidia. *Conidia* enteroarthric, rarely holoarthric, unicellular, hyaline, smooth- and thin-walled, mostly cylindrical, barrel-shaped, or irregularly shaped, detached from the fertile hyphae by rhexolysis. *Sexual morph* (when present) consisting in ascomata formed by of an anastomosing network of orange to brown, ornamented or not thick-walled hyphae (gymnothecia), bearing elongate appendages and/or spine projections, within there are small, evanescent, inflated asci which forms eight globose to oblate ascospores, whose cell wall is ornamented with a (coarse or thin) reticulate pattern. Species homothallic or heterothallic, thermotolerant or thermophilic, keratinolytic, chitinolytic or cellulolytic.

Taking into account that *Auxarthron* and *Malbranchea* are congeneric, as has been shown in previous studies (Sigler et al. 2002; Sarrocco et al. 2015) and here (Fig. 2), and that *Malbranchea* (Saccardo 1882) has historical priority (Turland et al. 2018) over *Auxarthron* (Orr et al. 1963), we transfer the species of *Auxarthron* to *Malbranchea* as follows:

***Malbranchea californiensis*** (G.F. Orr & Kuehn) Rodr.-Andr., Stchigel & Cano, **comb. nov.**

MycoBank MB 835229

*Basionym*: *Auxarthron californiense* G.F. Orr & Kuehn, *Can. J. Bot.* **41**: 1442 (1963).

*Synonym*: *Gymnoascus californiensis* (G.F. Orr & Kuehn) Apinis, *Mycol. Pap.* 96: 12 (1964).

***Malbranchea chinensis*** (Z.F. Zhang & L. Cai) Rodr.-Andr., Cano & Stchigel, **comb. nov.** MycoBank MB 839604

*Basionym*: *Auxarthron chinense* Z.F. Zhang & L. Cai, *Fungal Divers*. **106**: 55 (2020).

***Malbranchea chlamydospora*** (M. Solé et al.) Rodr.-Andr., Cano & Stchigel, **comb. nov*.***

MycoBank MB 835230

*Basionym*: *Auxarthron chlamydosporum* M. Solé, et al., *Stud. Mycol.* **47**: 108 (2002).

***Malbranchea compacta*** (G.F. Orr & Plunkett) Rodr.-Andr., Cano & Stchigel, **comb. nov.**

MycoBank MB 835231

*Basionym*: *Auxarthron compactum* G.F. Orr & Plunkett, *Can. J. Bot.* **41**: 1453 (1963).

***Malbranchea concentrica*** (M. Solé et al.) Rodr.-Andr., Stchigel & Cano, **comb. nov.**

MycoBank MB 835232

*Basionym*: *Auxarthron concentricum* M. Solé et al., *Stud. Mycol.* **47**: 106 (2002).

***Malbranchea conjugata*** (Kuehn) Rodr.-Andr., Cano & Stchigel, **comb. nov.**

MycoBank MB 835233

*Basionym*: *Myxotrichum conjugatum* Kuehn, *Mycologia* **47**: 883 (1956) [“1955”].

***Malbranchea guangxiensis*** (Z.F. Zhang & L. Cai) Rodr.-Andr., Cano & Stchigel, **comb. nov.**

MycoBank MB 839605

*Basionym*: *Auxarthron guangxiense* Z.F. Zhang & L. Cai, *Fungal Divers*. **106**: 57 (2020).

*Synonym*: *Auxarthron conjugatum* (Kuehn) G.F. Orr & Kuehn, *Mycotaxon* **24**: 148 (1985).

***Malbranchea longispora*** (Stchigel et al.) Rodr.-Andr., Stchigel & Cano, **comb. nov*.***

MycoBank MB 835235

*Basionym*: *Auxarthron longisporum* Stchigel et al., *Persoonia* **31**: 267 (2013).

***Malbranchea ostraviensis*** (Hubka et al.) Rodr.-Andr., Cano & Stchigel, **comb. nov*.***

MycoBank MB 835236

*Basionym*: *Auxarthron ostraviense* Hubka et al., *Med. Mycol.* **50**: 619 (2012).

***Malbranchea pseudauxarthron*** (G.F. Orr & Kuehn) Rodr.-Andr., Stchigel & Cano, **comb. nov.**

MycoBank MB835237

*Basionym*: *Auxarthron pseudauxarthron* G.F. Orr & Kuehn, *Mycologia* **64**: 67 (1972).

***Malbranchea umbrina*** (Boud.) Rodr.-Andr., Cano & Stchigel, **comb. nov.**

MycoBank MB 835238

*Basionym*: *Gymnoascus umbrinus* Boud., *Bull. Soc. mycol. Fr.* 8: **43** (1892).

*Synonyms*: *Auxarthron brunneum* (Rostr.) G.F. Orr & Kuehn, *Can. J. Bot.***41**: 1446 (1963).

*Auxarthron umbrinum* (Boud.) G.F. Orr & Plunkett, *Can. J. Bot.* **41**: 1449 (1963).

*Auxarthron thaxteri* (Kuehn) G.F. Orr & Kuehn, *Mycologia* **63**: 200 (1971).

*Gymnoascus subumbrinus* A.L. Sm. & Ramsb., *Trans. Br. Mycol. Soc*. **5**: 424 (1917) [“1916”].

*Gymnoascus umbrinus* var. *thaxteri* (Kuehn) Apinis, *Mycol. Pap.* 96: 14 (1964).

*Myxotrichum brunneum* Rostr., *Bot. Tidsskr.* **19**: 216 (1895).

*Myxotrichum thaxteri* Kuehn, *Mycologia* **47**: 878 (1956) [“1955”].

***Malbranchea zuffiana*** (Morini) Rodr.-Andr., Stchigel & Cano, **comb. nov.**

MycoBank MB 835239

*Basionym*: *Gymnoascus zuffianus* Morini, *Mem. R. Accad. Sci. Ist. Bologna*, ser. 4 **10**: 205 (1889).

*Synonym*: *Auxarthron zuffianum* (Morini) G.F. Orr & Kuehn, *Can. J. Bot.* **41**: 1445 (1963).

We also update the *Malbranchea* species names listed below:

***Malbranchea albolutea*** Sigler & J.W. Carmich., *Mycotaxon* **4**: 416 (1976).

*Synonym*: *Auxarthron alboluteum* Sigler et al., *Stud. Mycol.* **47**: 118 (2002).

***Malbranchea filamentosa*** Sigler & J.W. Carmich., *Mycotaxon* **15**: 468 (1982).

*Synonym*: *Auxarthron filamentosum* Sigler et al., *Stud. Mycol.* **47**: 116 (2002).

Because in a BLAST search using the ITS and LSU nucleotide sequences from the ex-type strains, *Malbranchea circinata* and *M. flavorosea* match with taxa in the family *Myxotrichaceae*, both those species are excluded to the genus.

After examination of the lectotype of *Auxarthron indicum* (Patil and Pawar 1987, as “*indica*”), we concluded that this fungus must be excluded from *Malbranchea* because its sexual morph differs mainly from all species described for the former genus. Whereas *Auxarthron indicum* produces smooth-walled ellipsoidal ascospores and gymnothecial ascomata lacking of true appendages, in *Malbranchea* spp. the ascospores are globose and mostly ornamented, and the ascomata have appendages. Based on the fact that there is no type strain of this species available we consider it as of uncertain application.

Despite the strain FMR 17681 being placed phylogenetically close to *Malbranchea ostraviense* and *M. umbrina*, it differs genetically and phenotypically from both species, therefore we describe the new species *Malbranchea gymnoascoides* as follows:

***Malbranchea gymnoascoides*** Rodr.-Andr., Stchigel & Cano, **sp. nov.**

**(**Fig. 6)

**Fig. 6:**
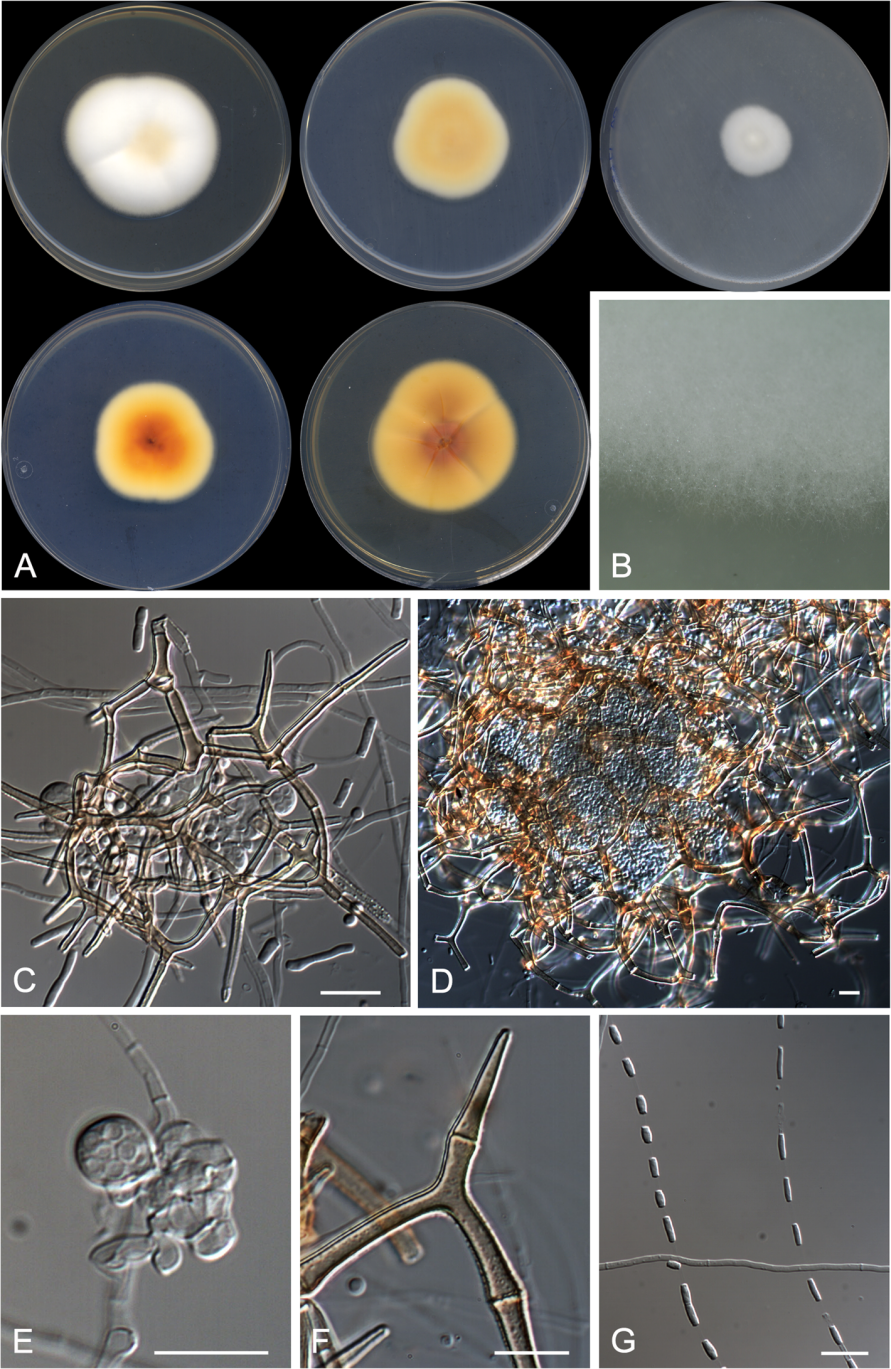
*Malbranchea gymnoascoides* CBS 146930 ^T^. **a** Colonies on PYE, PDA and OA after 14 d at 25 °C, from left to right (top row, surface; bottom row, reverse). **b** Detail of the colony on OA. **c**–**d** Young and mature ascomata. **e** Young ascus on fertile hyphae. **f** Peridial spine-like appendage. **g** Intercalary arthroconidia along the fertile hyphae. Scale bar = 10 μm

MycoBank MB 835212

*Etymology*: As the ascomata are morphologically like those of *Gymnoascus reessii.*

*Diagnosis*: *Malbranchea gymnoascoides* is phylogenetically close to *M. ostraviensis* and *M. umbrina* (Fig. 2). Nevertheless, *M. gymnoascoides* produces smaller ascomata (to 250 μm diam in *M. gymnoascoides* vs. to 450 and to 600 μm diam in both, *M. ostraviensis* and *M. umbrina*, respectively) (Orr et al. 1963; Hubka et al. 2013). Also, the peridial appendages of *M. gymnoascoides* are longer than those of *M. umbrina* (250–400 μm vs. 5–72 μm), but shorter than those of *M. ostraviensis* (350–600 μm long). The ascospores of *M. gymnoascoides* are like those of *M. ostraviensis* (smooth-walled under the bright field microscope, oblate to globose, 2.5–3.5 μm diam), whereas those of *M. umbrina* are lenticular and measure 2.8–4.0 × 2.1–2.6 μm. Moreover, the arthroconidia of *M. gymnoascoides* are larger than those of *M. umbrina* (6.0–10.0 × 1.5–2.0 μm and 2.6–7.0 × 1.4 μm, respectively). *Malbranchea ostraviensis* also produces a pinkish to red diffusible pigment on MEA, PDA and SDA, a feature not observed in *M. gymnoascoides* nor in *M. umbrina*. Both *Malbranchea gymnoascoides* as well as of *M. umbrina* can grow slowly at 35 °C, whereas the maximum temperature of growth for *M. ostraviensis* is of 32 °C.

*Type*: **USA**: *Texas*: from human bronchial washing specimen, 2005, *N. Wiederhold* (CBS H-24456 – holotype; CBS 146930 = FMR 17681 = UTHSCSA DI18-87 – ex-type cultures; LSU/ITS sequences GenBank LR701758/LR701757).

*Description: Vegetative hyphae* septate, hyaline, smooth- and thin-walled, mostly straight, rarely branched, 1.5–2.5 μm wide. *Asexual morph* consisting in undifferentiated *fertile hyphae* which form randomly intercalary and terminally arthroconidia. *Conidia* enteroarthric, unicellular, hyaline, smooth- and thin-walled, mostly barrel-shaped, sometimes cylindrical or irregularly-shaped, 6.0–10.0 × 1.5–2.0 μm, detached by rhexolysis. *Ascomata* gymnothecial, solitary or in clusters, hyaline at first, becoming orange brown with the age, globose or nearly so, 130–250 μm diam excluding the appendages, which cover entirely the surface. *Peridial hyphae* septate, orange brown, branching and anastomosing to form a reticulate network, asperulate, very thick-walled, 3.5–5.5 μm wide, fragmenting by the septa when ageing, with lateral appendages. *Appendages* 0–1-septate, orange brown, asperulate, thick-walled, progressively tapering towards the apex, apex sinuous, 250–400 μm long, connected by basal knuckle joints. *Asci* 8-spored, globose or nearly so, 4–7 μm diam, soon deliquescent. *Ascospores* unicellular, hyaline at first, yellowish in mass when mature, smooth-walled under bright field microscope, globose, 2.5–3.5 μm diam.

*Culture characteristics*: Colonies on PYE reaching 46–47 mm diam after 2 wk. at 25 °C, slightly elevated, velvety to floccose, margins regular, pale orange (5A3) at centre and white (5A1) at edge, sporulation sparse; reverse orange (5A6). Colonies on PDA reaching 36–37 mm diam after 2 wk. at 25 °C, slightly elevated, velvety, margins regular, light orange (5A5) at centre and orange white (5A2) at edge, sporulation sparse; reverse deep orange (6A8). Colonies on PDA reaching 31–32 mm diam after 2 wk. at 30 °C, slightly elevated, velvety, margins regular, slightly furrowed, orange (5A6), sporulation sparse; reverse brownish orange (6C8). Colonies on OA reaching 21–22 mm diam after 2 wk. at 25 °C, slightly elevated, velvety, margins regular, orange white (5A2) at centre and white (5A1) at edge, sporulation sparse. Exudate and diffusible pigment absent in all culture media tested. Minimum, optimal and maximum temperature of growth on PDA: 10 °C, 25 °C, and 35 °C, respectively. Non-haemolytic. Casein hydrolysed without pH change. Not inhibited by cycloheximide. Urease and esterase tests positive. Growth occurs at NaCl 10% w/w, but not at 20% w/w.

Despite the strain FMR 17695 being phylogenetically close to *Malbranchea longispora*, it differs phylogenetically and morphologically from it. Consequently, we describe the new species *Malbranchea multiseptata*.

***Malbranchea multiseptata*** Rodr.-Andr., Cano & Stchigel, **sp. nov.**

(Fig. 7)

**Fig. 7:**
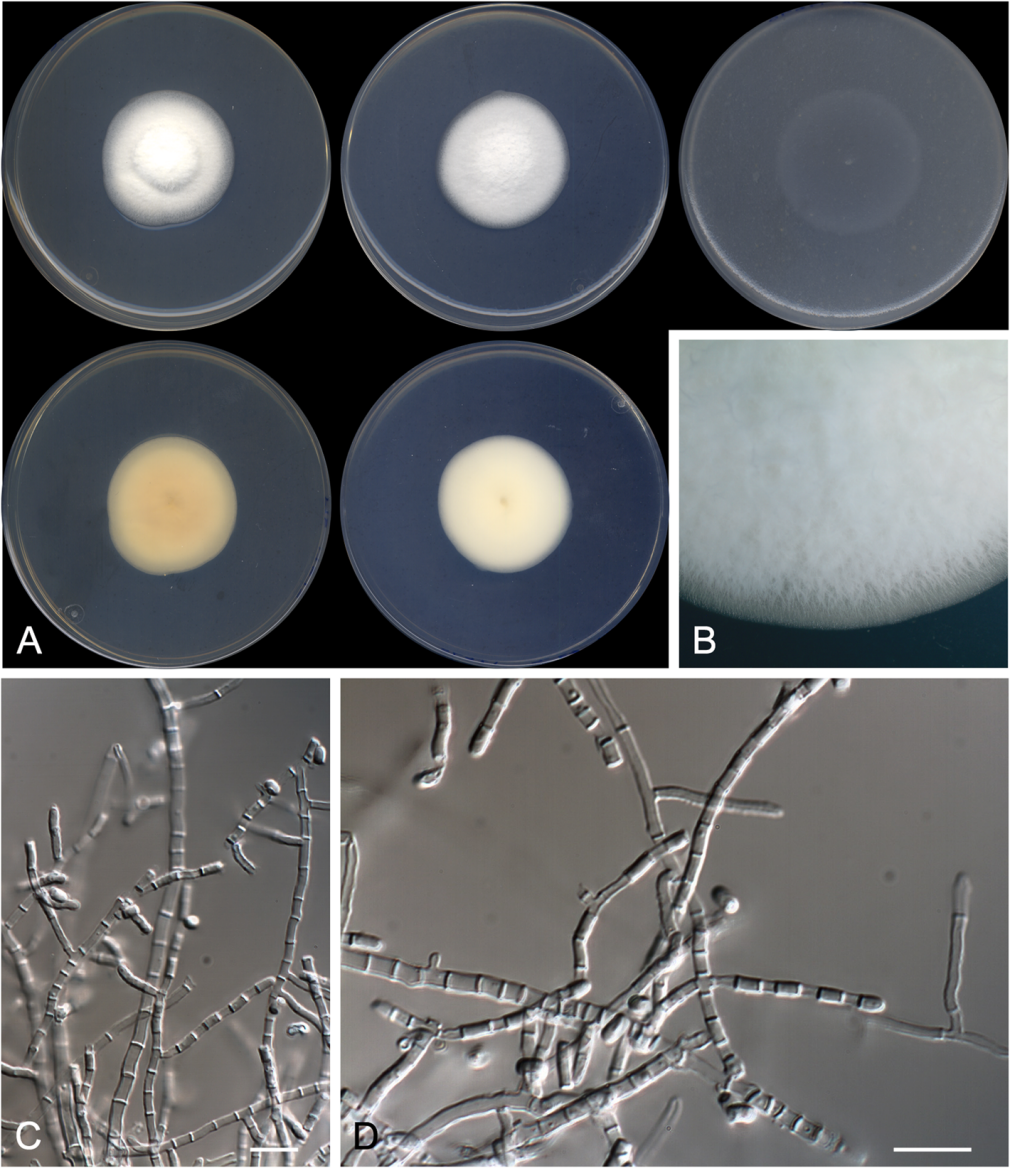
*Malbranchea multiseptata* CBS 146931 ^T^. **a** Colonies on PYE, PDA and OA after 14 d at 25 °C, from left to right (top row, surface; bottom row, reverse). **b** Detail of the colony on PDA. **c**–**d** Highly septate fertile hyphae and arthroconidia. Scale bar = 10 μm

MycoBank MB 835213

*Etymology*: From Latin *multi*-, many, and –*septatae*, septa, because the vegetative hyphae are multiseptate.

*Diagnosis*: *Malbranchea multiseptata* is phylogenetically linked to *M. longispora*. Nevertheless, *M. multiseptata* does not form chlamydospores nor a sexual morph as in *M. longispora* (Crous et al. 2013). Also, *M. multiseptata* produces shorter conidia (3.0–9.0 × 1.5–2.0 μm) than those of *M. longispora* (4.0–24.0 × 1.0–5.5 μm).

*Type*: **USA**: *Texas*: from human bronchial washing specimen, 2014, *N. Wiederhold* (CBS H-24457 – holotype; CBS 146931 = FMR 17695 = UTHSCSA DI18-101 – ex-type cultures; LSU/ITS sequences GenBank LR701760/LR701759).

*Description: Vegetative hyphae* hyaline, smooth- and thin-walled straight to sinuous, sparsely branched, 1.0–2.0 μm wide, becoming highly septate with the age, septa thickened. *Fertile hyphae* arising as lateral branches (sometimes opposite each other) from the vegetative hyphae, unbranched, straight or slightly sinuous, 1.5–2.0 μm wide, forming randomly intercalary and terminally arthroconidia. *Conidia* enteroarthric, unicellular, hyaline, smooth- and thin-walled, separated by evanescent connective cells, cylindrical, 3.0–9.0 × 1.5–2.0 μm, rounded at the end when terminal, rhexolytic secession. *Chlamydospores*, *racquet hyphae*, *setae*, and *sexual morph* not observed.

*Culture characteristics*: Colonies on PYE reaching 35–36 mm diam after 2 wk. at 25 °C, elevated, velvety to floccose, margins regular, white (5A1), sporulation sparse; reverse greyish yellow (4B4). Colonies on PDA reaching 34–35 mm diam after 2 wk. at 25 °C, slightly elevated, velvety to floccose, margins regular, white (5A1), sporulation absent; reverse yellowish white (3A2). Colonies on PDA reaching 27–28 mm diam after 2 wk. at 30 °C, slightly elevated, velvety to floccose, margins regular, white (5A1), sporulation absent; reverse pale yellow (3A3). Colonies on OA researching 37–38 mm diam after 2 wk. at 25 °C, flattened, barely perceptible growth, not distinguishable colour, sporulation sparse. Exudate and diffusible pigment absent in all culture media tested. Minimum, optimal and maximum temperature of growth on PDA: 10 °C, 25 °C, and 35 °C, respectively. Haemolytic. Casein hydrolyzed without pH change. Not inhibited by cycloheximide. Urease positive. Growth occurs at NaCl 3% w/w, but not at 10%w/w. Neither grow on TOTM.

Because the strain FMR 17680 was placed phylogenetically close to *Malbranchea filamentosa* but in a separate terminal branch, and because both differ morphologically and genotypically, the new species *Malbranchea stricta* is also described*.*

***Malbranchea stricta*** Rodr.-Andr., Stchigel & Cano, **sp. nov.**

**(**Fig. 8)

**Fig. 8:**
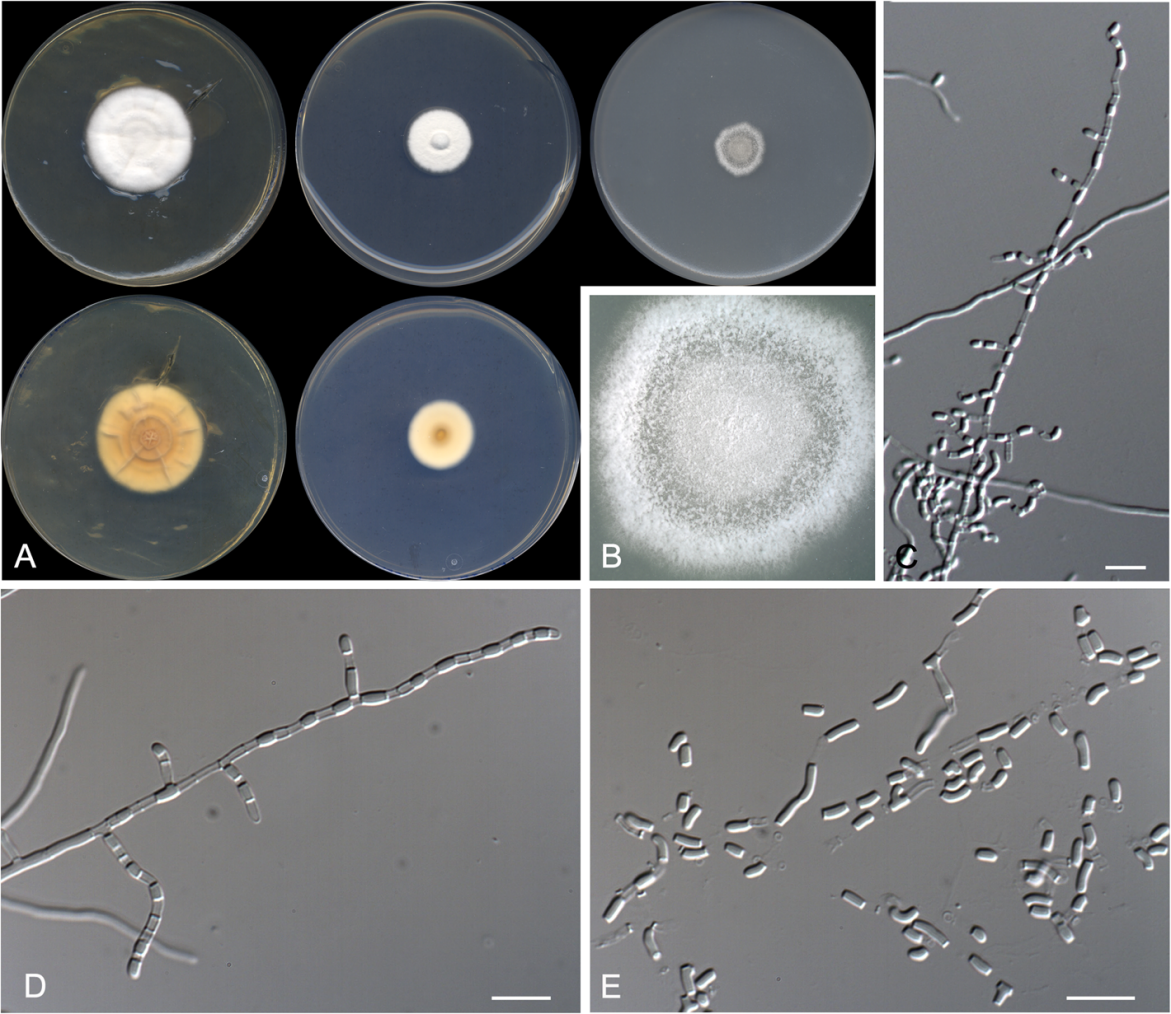
*Malbranchea stricta* CBS 146932 ^T^. **a** Colonies on PYE, PDA and OA after 14 d at 25 °C, from left to right (top row, surface; bottom row, reverse). **b** Detail of the colony on OA. **c**–**e** Alternate arthroconidia on primary hyphae and lateral branches. Scale bar = 10 μm

MycoBank MB 835219

*Etymology:* Latin *stricta*, strict, due to the production of the typical reproductive structures of the genus.

*Diagnosis*: *Malbranchea stricta* is phylogenetically close to *M. filamentosa*. Also, both species lack a sexual morph (Sigler et al. 2002). However, *M. filamentosa* produces more regularly shaped conidia than *M. stricta*, and forms thick-walled brown setae, structures absent in *M. stricta*.

*Type*: **USA**: *Florida*: human nail, 2003, *N. Wiederhold* (CBS H-24458 – holotype; CBS 146932 = FMR 17680 = UTHSCSA DI18-86 – ex-type cultures; LSU/ITS sequences GenBank LR701639/LR701638).

*Description: Vegetative hyphae* hyaline, smooth- and thin-walled, straight to sinuous, sparsely branched, 1.5–2.0 μm wide. *Fertile hyphae* well-developed, arising as lateral branches from the vegetative hyphae, mostly unbranched, right or slightly sinuous, contorted or arcuate at the end, up to 25 μm long, 1.5–2.0 μm wide, or developing at the extremes of the vegetative hyphae, in both cases forming arthroconidia randomly intercalary and terminally. *Arthroconidia* enteroarthric, hyaline, becoming yellowish with the age, barrel-shaped, “T”-shaped, “Y”-shaped, finger-shaped or irregularly-shaped, 2.0–6.0 × 1.0–2.0 μm, with rhexolytic secession. *Chlamydospores*, *racquet hyphae*, and *sexual morph* not observed.

*Culture characteristics: Colonies* on PYE reaching 32–33 mm diam after 2 wk. at 25 °C, flattened, velvety, regular margins, furrowed, white (4A1), sporulation sparse; reverse pale orange (5A3). Colonies on PDA reaching 20–21 mm diam after 2 wk. at 25 °C, slightly elevated, velvety to floccose, regular margins, white (3A1), sporulation abundant; reverse pale yellow (4A3). Colonies on PDA reaching 20–21 mm diam after 2 wk. at 30 °C, slightly elevated, velvety to floccose, margins regular, white (3A1), sporulation abundant; reverse yellowish brown (5E8) at centre and greyish yellow (4B5) at the margins. Colonies on OA researching 16–17 mm diam after 2 wk. at 25 °C, flattened, granulose, white (3A1), margins regular, sporulation sparse. Exudate and diffusible pigment absent. Minimum, optimum and maximum temperature of growth on PDA: 10 °C, 30 °C, and 37 °C, respectively. Colonies haemolytic (on BA), and casein hydrolyzed without pH changes at 25 °C (on BCP-MS-G). Not inhibited by cycloheximide. Urease and esterase tests positive. Growth occurs at NaCl 10% w/w, but not at 20% w/w.

***Pseudoarthropsis***

Since the strain FMR 17692 was placed in the same terminal clade as *Arthropsis cirrhata*, while the type species of the genus (*Arthropsis truncata*) is phylogenetically distant (in *Sordariales*; Giraldo et al. 2013), we erect the new genus *Pseudoarthropsis* for *A. cirrhata*, and the new species *Pseudoarthropsis crassispora*.

***Pseudoarthropsis*** Stchigel, Rodr.-Andr. & Cano, **gen. nov.**

MycoBank MB 834925

*Etymology:* From Greek *ψευδής*-, resembling, because the morphological semblance to *Arthropsis*.

*Diagnosis*: *Mycelium* composed by hyaline to orange, septate hyphae. *Conidiophores* consisting of fertile lateral branches and a portion of the main subtending hypha, with all these structures disintegrating into yellowish orange, thin-walled, cylindrical to cuboid enteroarthric conidia, or into hyaline, thick-walled, ellipsoidal, globose to barrel-shaped holoarthric conidia.

*Type species: Pseudoarthropsis cirrhata* (Oorschot & de Hoog) Stchigel, Rodr.-Andr. & Cano 2021.

***Pseudoarthropsis cirrhata*** (Oorschot & de Hoog) Stchigel, Rodr.-Andr. & Cano, **comb. nov.** MycoBank MB 834928

*Basionym*: *Arthropsis cirrhata* Oorschot & de Hoog, *Mycotaxon* **20**: 130 (1984).

*Description: Vegetative hyphae* septate, pale yellowish orange, smooth- and thin-walled, dichotomously branched, 2–3 μm wide. *Fertile hyphae* well differentiated, arising at right angles as recurved lateral branches of the vegetative hyphae, forming septa basipetally to produce chains of enteroarthric conidia. *Arthroconidia* yellowish orange, smooth- and thin-walled, cylindrical to cuboid, often broader than long, 2.5–4.0 × 2–3 μm, truncated at both ends, separated by trapezoid connectives, secession rhexolytic. *Colonies* on PYE reaching 4–5 mm diam after 10 d at 25 °C, powdery, fealty, slightly raised, orange (5A7), pale orange (5A5) at centre; reverse brownish orange (7C8), diffusible pigment brown.

*Type*: **The Netherlands**: from a wall near Schiphol, 1984, *C.A.N. van Oorschot* (CBS 628.83).

***Pseudoarthropsis crassispora*** Rodr.-Andr., Stchigel & Cano*,* **sp. nov*.***

**(**Fig. 9)

**Fig. 9:**
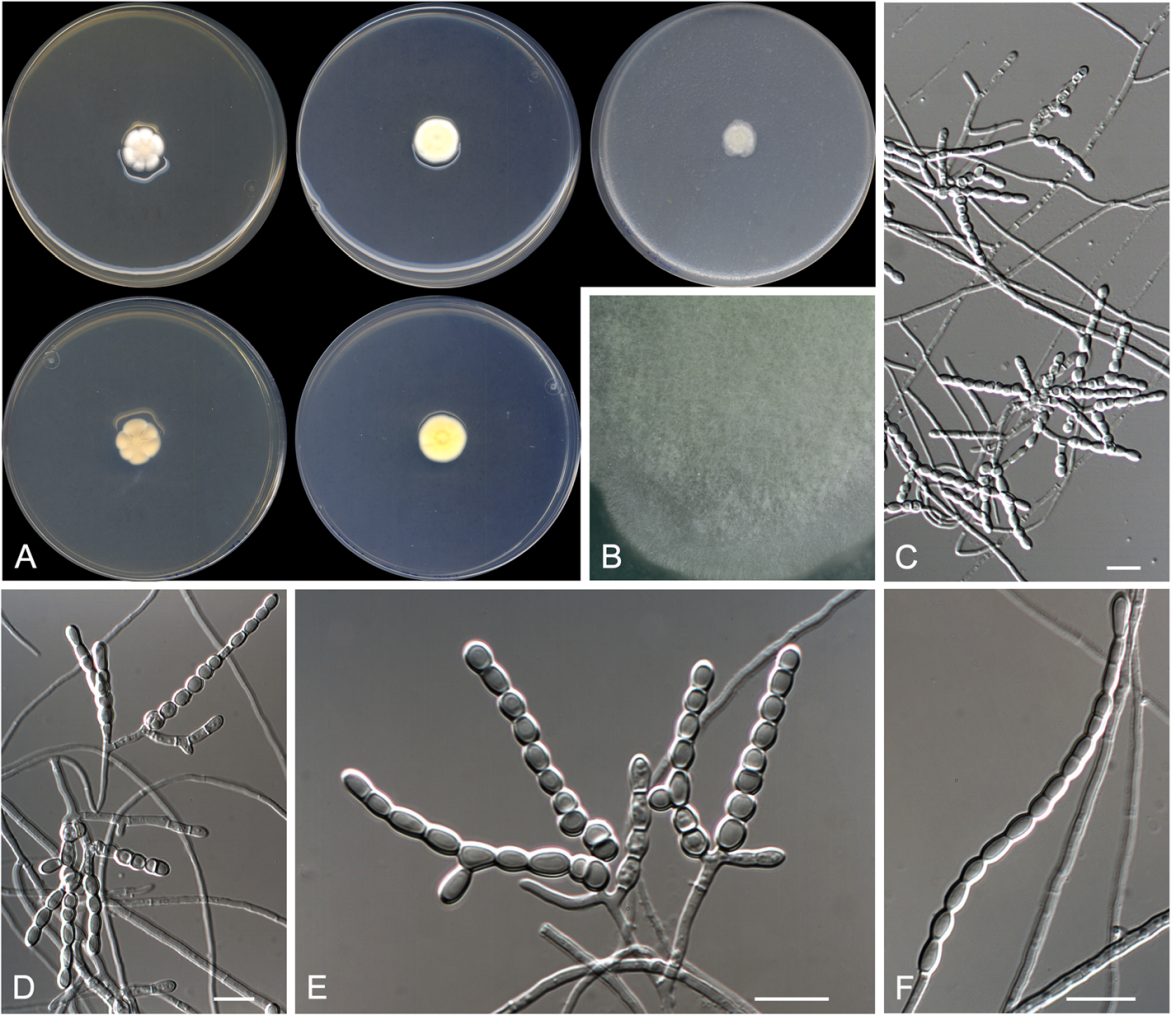
*Pseudoarthropsis crassispora* CBS 146928 ^T^. **a** Colonies on PYE, PDA and OA after 14 d at 25 °C, from left to right (top row, surface; bottom row, reverse). **b** Detail of the colony on OA. **c**–**e** Bi- to trichotomously-branched fertile hyphae. **f** A large chain of holoarthric conidia. Scale bar = 10 μm

MycoBank MB 834930

*Etymology*: From Latin *crassus*-, thick, and -*sporarum*, spore, because of the thick wall of the conidia.

*Diagnosis*: *Pseudoarthropsis crassispora* is phylogenetically close to *P. cirrhata*. Nevertheless, the former produces holoarthric conidia, while they are enteroarthric in the latter. Also, the conidia of *P. crassispora* are ellipsoidal, globose or broadly barrel-shaped, while these are cylindrical to cuboid (often wider than they are long) in *P. cirrhata* (van Oorschot and de Hoog 1984). Moreover, the conidia are bigger in *P. crassispora* than in *P. cirrhata* (4.5–5.5 × 2.5–3.5 μm vs. 2.5–4.0 × 2.0–3.0 μm). Also, *P. crassispora* grows faster than *P. cirrhata* (on PYE at 25 °C), and the maximum temperature of growth is at 37 °C and 30 °C, respectively.

*Type*: **USA**: *Minnesota*: from a human bronchial washing specimen, 2012, *N. Wiederhold* (CBS H-24454 – holotype; CBS 146928 = FMR 17692 = UTHSCSA DI18-98 – ex-type cultures; LSU/ITS sequences GenBank LR701763/LR701764).

*Description: Vegetative hyphae* septate, hyaline, smooth- and thin-walled, mostly straight, occasionally branched, 1.5–2.0 μm wide. *Fertile hyphae* well-differentiated, arising as lateral branches of the vegetative hyphae, hyaline, septate, smooth- and thin-walled, erect, simple or branched up to 3 times at the apex, stipe 10–20 × 1.5–2.0 μm, branches 10–70 × 1.5–2.0 μm, forming septa basipetally to produce chains of arthroconidia. *Conidia* holoarthric, unicellular, hyaline, smooth- and thick-walled, ellipsoidal, globose or barrel-shaped, transiently presents as bi-cellular conidia, 2.5–3.5 × 4.5–5.5 μm, in chains of up to 20, separate from the fertile hyphae by schizolysis, rarely by rhexolysis. *Chlamydospores*, *racquet hyphae*, *setae*, and *sexual morph* not observed.

*Culture characteristics*: Colonies on PYE reaching 13–14 mm diam after 2 wk. at 25 °C, slightly elevated, velvety, margins regular, furrowed, yellowish white (3A2) and yellowish grey (4B2) at centre, sporulation abundant; reverse pale yellow (4A3. Colonies on PDA reaching 14–15 mm diam after 2 wk. at 25 °C, flattened, velvety, margins regular, greenish white (30A2) and pastel green (30A4) at centre, sporulation abundant; reverse pastel yellow (3A4). Colonies on PDA reaching 15–16 mm diam after 2 wk. at 30 °C, slightly elevated, velvety, margins regular, furrowed, yellowish white (3A2), sporulation sparse; reverse yellow (3A6), with a scarce production of yellowish diffusible pigment. Colonies on OA researching 10–11 mm diam after 2 wk. at 25 °C, flattened, velvety to floccose, margins irregular, greenish white (30A2) and pale green (28A3) at centre, sporulation abundant. Exudate and diffusible pigment absent, except on PDA. Minimum, optimal and maximum temperature of growth on PDA: 10 °C, 30 °C, and 37 °C, respectively. Non-haemolytic. Casein hydrolyzed without pH change. Not inhibited by cycloheximide. Urease and esterase tests positive. The fungus grows up to NaCl 10% w/w, but not at 20% w/w.

***Pseudomalbranchea***

Despite the strain FMR 17684 being placed phylogenetically in *Onygenaceae*, it is paraphyletic described as the type species of the new genus *Pseudomalbranchea*.

***Pseudomalbranchea*** Rodr.-Andr., Cano & Stchigel, **gen. nov.**

MycoBank MB 835220

*Etymology:* Recalling the morphological similarity with *Malbranchea*.

*Diagnosis*: *Arthroconidia* one-celled, intercalary disposed along unbranched vegetative hyphae, mostly enteroarthric, occasionally holoarthric, cylindrical but becoming globose with the age.

*Type species*: ***Pseudomalbranchea gemmata*** Rodr.-Andr., Cano & Stchigel 2021

*Description: Mycelium* sparse, composed of hyaline, smooth- and thin-walled septate hyphae. *Asexual morph* consisting of mostly enteroarthric, occasionally holoarthric, conidia, intercalary disposed along unbranched vegetative hyphae, solitary or in short chains, with rhexolytic or rarely schizolytic secession. *Arthroconidia* one-celled, hyaline, smooth- and thick-walled, cylindrical but becoming globose with the age. *Chlamydospores, racquet hyphae* and s*exual morph* not observed.

***Pseudomalbranchea gemmata*** Rodr.-Andr., Cano & Stchigel, **sp. nov.**

(Fig. 10)

**Fig. 10:**
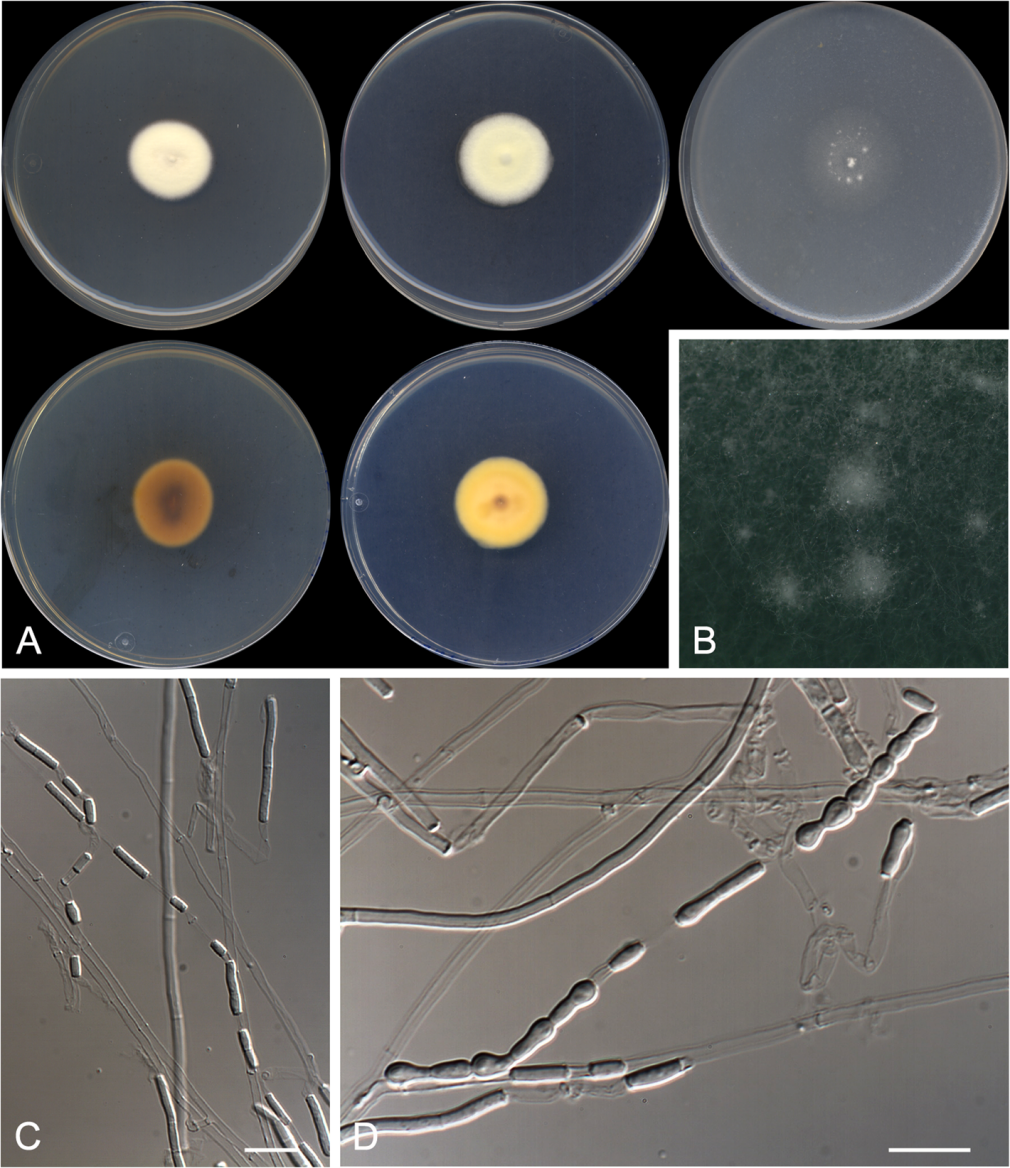
*Pseudomalbranchea gemmata* CBS 146933 ^T^. **a** Colonies on PYE, PDA and OA after 14 d at 25 °C, from left to right (top row, surface; bottom row, reverse). **b** Detail of the colony on OA. **c**–**d** Large, intercalary, irregularly-shaped arthroconidia disposed singly or in chains along the fertile hyphae. Scale bar = 10 μm

MycoBank MB 835221

*Etymology:* From the Latin *gemmatum*, jewelled, because the swollen conidia disposed in chains.

*Diagnosis*: *Pseudomalbranchea gemmata* is phylogenetically close to *Uncinocarpus reesii* and *Amauroascus volatilis-patellis*. However, it does not produce a sexual morph and it differs from *U. reessi* and *A. volatilis-patellis* by the production of longer arthroconidia (4.0–11.0 × 2.0–3.5 μm in *P. gemmata* vs. 3.5–6.0 × 2.5–3 μm in *U. reessi*, and 4.0–5.4 × 2.0–3.0 in *A. volatilis-patellis*; Orr and Kuehn 1972, Sigler and Carmichael 1976, Currah 1985). As well as *A. volatilis-patellis*, *P. gemmata* lacks appendages, which are present and similar to the asexual morph in *U. reessi* (Currah 1985).

*Type*: **USA**: *Florida*: from human bronchial washing specimen, 2014, *N. Wiederhold* (CBS H-24459 – holotype, CBS 146933 = FMR 17684 = UTHSCSA DI18-90 – ex-type cultures; LSU/ITS sequences GenBank LR701762/LR701761).

*Description: Mycelium* sparse, composed of hyaline, smooth- and thin-walled, sparsely septate hyphae, 1.0–2.0 μm wide. *Conidia* enteroarthric (occasionally holoarthric), intercalary disposed along unbranched vegetative hyphae, one-celled, solitary or in short chains of up to 7, one-celled, hyaline, smooth- and thick-walled, cylindrical but becoming globose with the age, 4.0–11.0 × 2.0–3.5 μm, liberated from the fertile hyphae by rhexolysis (rarely by schizolysis). *Chlamydospores*, *racquet hyphae* and *sexual morph* not observed.

*Culture characteristics*: Colonies on PYE reaching 22–23 mm diam after 2 wk. at 25 °C, slightly elevated, velvety, margins regular, pale yellow (3A3), sporulation sparse; reverse brown (6E6). Colonies on PDA reaching 24–25 mm diam after 2 wk. at 25 °C, slightly elevated, velvety, margins regular, pale yellow (3A3), sporulation sparse; reverse light yellow (4A5). Colonies on PDA reaching 25–26 mm diam after 2 wk. at 30 °C, flattened, radially folded, velvety, margins regular, pale yellow (3A3), sporulation sparse; reverse light yellow (4A5). Colonies on OA reaching 28–29 mm diam after 2 wk. at 25 °C, flattened, velvety to granulose, irregular margins, white (6A1), sporulation sparse. Exudate and diffusible pigment lacking*.* Minimum, optimum and maximum temperature of growth on PDA: 10 °C, 30 °C, and 37 °C, respectively. Colonies haemolytic, casein not hydrolyzed. The fungus was not inhibited by cycloheximide. Urease and esterase tests positive. Growth occurs at NaCl 3% w/w, but not higher concentration.

***Spiromastigoides***

Because strains FMR 17686 and FMR 17696 were placed together in a terminal branch close to the ex-type strain of *M. gypsea* in the *Spiromastigaceae* clade (Fig. 2), *M. gypsea* is combined into *Spiromastigoides* and these two strains are described as the new species *S. geomycoides*.

***Spiromastigoides geomycoides*** Stchigel, Rodr.-Andr. & Cano, **sp. nov.**

**(**Fig. 11)

**Fig. 11:**
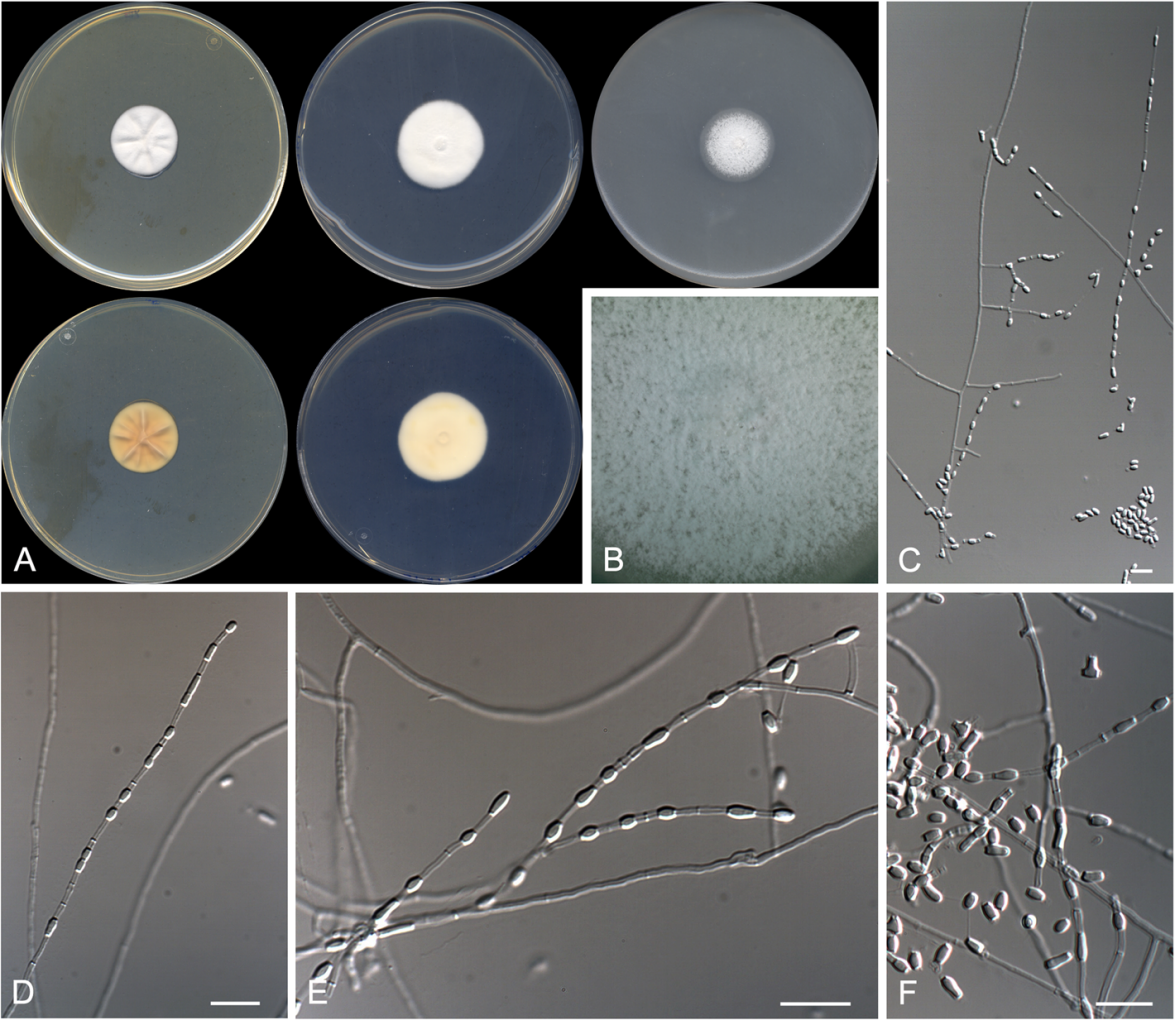
*Spiromastigoides geomycoides* CBS 146934 ^T^. **a** Colonies on PYE, PDA and OA after 14 d at 25 °C, from left to right (top row, surface; bottom row, reverse). **b** Detail of the colony on OA. **c** Fertile lateral branches mimicking *Geomyces* spp. conidiophores. **d**–**e** Fertile hyphae with intercalary, barrel-shaped arthroconidia. **f** Morphological diversity of arthroconidia. Scale bar = 10 μm

MycoBank MB 835222

*Etymology:* From the production of conidiophores morphologically similar to those of the genus *Geomyces*.

*Diagnosis*: *Spiromastigoides geomycoides* is phylogenetically close to *S. gypsea*. However, it produces smaller conidia (1.5–2.5 × 1.0–2.0 μm) than *S. gypsea* [(2.5)3–6(9) × 2–2.5 μm; Sigler and Carmichael 1976]. Also, *S. geomycoides* grows faster than *S. gypsea* on PYE at 35 °C.

*Type:***USA**: *Illinois*: from a human foot skin, 2014, *N. Wiederhold* (CBS H-24460 – holotype, CBS 146934 = FMR 17696 = UTHSCSA DI18-102 – ex-type cultures; LSU/ITS sequences GenBank LR701768/LR701768).

*Description: Mycelium* abundant, composed of hyaline, smooth- and thin-walled, septate, branched, 1.0–2.0 μm wide hyphae, septa thickened with age. *Fertile hyphae* arising as lateral branches, straight or slightly curved, unbranched or, rarely, with a branching pattern similar to that of the conidiophores of *Geomyces*, septate, hyaline, smooth- and thin-walled, producing intercalary and terminally arthroconidia separated by 1–2 empty intermediary cells. *Conidia* enteroarthic, unicellular, hyaline, mostly barrel-shaped, less frequently “T”-shaped or cylindrical, 1.5–2.5 × 1.0–2.0 μm, rhexolytic dehiscence. *Chlamydospores*, *racquet hyphae* and *sexual morph* not observed.

*Culture characteristics*: Colonies on PYE reaching 24–25 mm diam after 2 wk. at 25 °C, flattened, velvety, furrowed, regular margins, white (4A1), abundant sporulation; reverse, pale orange (5A3). Colonies on PDA reaching 26–27 mm diam after 2 wk. at 25 °C, flattened, velvety, regular margins, white (4A1), abundant sporulation; reverse, yellowish white (4A2). Colonies on PDA reaching more than 90 mm diam after 2 wk. at 30 °C, flattened, velvety, regular margins, yellowish white (4A2), sporulation absent; reverse, pale yellow (4A3). Colonies on OA researching 20–21 mm diam after 2 wk. at 25 °C, flattened, granulose, regular margins, white (4A1), abundant sporulation. Exudate and diffusible pigment absent in all culture media tested. Minimum, optimum and maximum temperature of growth on PDA: 5 °C, 30 °C, and 37 °C, respectively. Colonies non-haemolytic. Casein not hydrolyzed. Resistant to cycloheximide. Urease negative and esterase positive. Growth occurs at NaCl 10% w/w, but not at 20% w/w.

*Other specimen examined*: **USA**: *Minnesota*: from blood, 2009, *N. Wiederhold* (FMR 17686).

**Spiromastigoides gypsea** (Sigler & Carmichael) Stchigel, Rodr.-Andr. & Cano, **comb. nov.**

MycoBank MB 835228

Basionym: Malbranchea gypsea Sigler & Carmichael, Mycotaxon **4**: 455 (1976).

Description (adapted from the original description): Arthroconidia produced intercalary or terminally along straight primary hyphae, or on short or long lateral branches, separated each one by one or more alternate empty cells, or, rarely, formed immediately adjacent to each other. Arthroconidia unicellular, hyaline, smooth- and thin-walled, cylindrical or slightly barrel-shaped, (2.5) 3–6 (9) × 2–2.5 μm, slightly broader than the interconnecting cells. No sexual morph obtained by matting. Colonies on PYE reaching 17–39 mm after three wk. at room temperature, chalky white to creamy white, downy to velvety, slightly raised, surface folded to convoluted, umbonated at centre, reverse buff. Optimum temperature of growth 25–30 °C. Maximum temperature of growth 37 °C (but strain depending).

### KEYS

***Key to*** **Arachnomyces** ***species***

Adapted from Sun et al. (2019).

1 Homothallic; asexual morph present or not.................................................................................................................................. 2

Heterothallic; asexual morph present ................................................................................................................................................. 6

2(1) Peridial setae coiled or circinate; asexual morph absent....................................................................................................... 3

Peridial setae straight, tapering towards the apex; asexual morph arthroconidia ……………................................. **gracilis**

3(2) Peridial setae slightly nodose; ascospores mostly < 3.5 μm diam ………………….…...................................................... 4

Peridial setae smooth-walled; ascospores mostly > 3.5 μm diam................................................................................................. 5

4(3) Ascospores smooth-walled............................................................................................................................................. **minimus**

Ascospores echinulate.......................................................................................................................................................... **peruvianus**

5(3) Ascomata 100–300 μm diam............................................................................................................................................. **nitidus**

Ascomata 500–700 μm diam.............................................................................................................................................. **sulphureus**

6(1) Arthroconidia alternate................................................................................................................................................................... 7

Arthroconidia in persistent chains..................................................................................................................................................... 12

7(6) Arthroconidia cylindrical or barrel-shaped; sclerotia present............................................................................................. 8

Arthroconidia distinct; sclerotia absent.............................................................................................................................................. 9

8(7) Colonies becoming greyish brown, not growing at 35 °C..................................................................................... **glareosus**

Colonies white to pale brown, growing at 35 °C........................................................................................................... **scleroticus**

9(7) Arthroconidia subglobose to pyriform..................................................................................................................................... 10

Arthroconidia cylindrical to finger-like-shaped.............................................................................................................................. 11

10(9) Arthroconidia smooth-walled to finely asperulate; setae (produced on the vegetative mycelium) smooth-walled to slightly nodose............................................................................................................................... **kanei**

Mature arthroconidia coarsely verrucose; setae (produced on the vegetative mycelium) strongly nodose............................................................................................................................................................. **pilosus**

11(9) Fertile hyphae successively branching to form dense clusters, arcuate, sinuous, contorted or tightly curled................................................................................................................ **bostrychodes**

Fertile hyphae branching but not in clusters; branches only apically coiled.......................................................................... **graciliformis**

12(6) Setae (produced on the vegetative mycelium) strongly nodose, circinate or loosely coiled at the apex................................................................................................................................................ **nodosetosus**

Setae (produced on the vegetative mycelium) strongly nodose, tip straight................................................................... **jinanicus**

***Key to*** **Malbranchea** ***species***

Adapted from Sigler and Carmichael (1976), Solé et al. (2002), and Hubka et al. (2013).

1 Homothallic species.............................................................................................................................................................................. 2

Heterothallic species............................................................................................................................................................................... 13

2(1) Peridial appendages longer than 150 μm long.......................................................................................................................... 3

Peridial appendages shorter or absent................................................................................................................................................. 8

3(2) Appendages 350–600 μm in length; diffusible pigment pinkish to reddish; not growing at 35 °C …............ **ostraviensis**

Above features not combined................................................................................................................................................................ 4

4(3) Ascospores smooth-walled under bright field microscope....................................................................... **gymnoascoides**

Ascospores reticulate................................................................................................................................................................................ 5

5(4) Peridial cells short, 4–12 μm in length; peridial projections with truncate ends.......................................... **compacta**

Peridial cells longer; peridial projections with mostly acute ends............................................................................................... 6

6(5) Ascospores usually exceeding 4 μm diam........................................................................................................*.* **californiensis**

Ascospores ≤4 μm diam........................................................................................................................................................................... 7

7(6) Species growing at 37 °C................................................................................................................................................ **conjugata**

No growth at 37 °C..................................................................................................................................................................... **umbrina**

8(2) Asexual morph not produced......................................................................................... **guangxiensis** / **pseudauxarthron**

Malbranchea-like asexual morph present........................................................................................................................................... 9

9(8) Ascomata with spine-like peridial projections, 27–40 μm in length.................................................................... **zuffiana**

Ascomata without peridial projections............................................................................................................................................. 10

10(9) Colonies on PDA brown................................................................................................................................................. **kuehnii**

Colonies on PDA otherwise................................................................................................................................................................. 11

11(10) Peridial hyphae smooth-walled........................................................................................................................... **concentrica**

Peridial hyphae strongly ornamented; chlamydospores present ….......................................................................................... 12

12(11) Arthroconidia 2–10 × 2.5–3.5 μm; growing above 30 °C …................................................................. **chlamydospora**

Arthroconidia 4–24 × 1.0–5.5 μm; not growing above 30 °C..................................................................................... **longispora**

13(1) Fertile hyphae arcuate or curved............................................................................................................................................. 14

Fertile hyphae straight to sinuous, branched or not..................................................................................................................... 21

14(13) Fertile hyphae coiled................................................................................................................................................................. 15

Fertile hyphae curved or arcuate........................................................................................................................................................ 16

15(14) Thermophilic; conidia 2.5–4.5 μm wide......................................................................................................... **cinnamomea**

Not thermophilic; conidia narrower.................................................................................................................................... **pulchella**

16(14) Colonies orange.......................................................................................................................................................................... 17

Colonies different.................................................................................................................................................................................... 18

17(16) Aleuroconidia laterally or terminally dispersed.................................................................................... **chrysosporoidea**

Aleuroconidia absent............................................................................................................................................................. **aurantiaca**

18(16) Colonies golden yellow, exudate brown, diffusible pigment yellow........................................................ **graminicola**

Above features not combined..................................................... .........................................................................................................19

19(18) Sexual morph produced by in vitro mating of compatible strains................................................................ **albolutea**

Sexual morph not formed..................................................................................................................................................................... 20

20(19) Thick-walled brown setae produced on OA from the vegetative mycelium......................................... **filamentosa**

Setae not produced....................................................................................................................................................................... **arcuata**

21(13) Fertile hyphae unbranched or scarcely branched............................................................................................................. 22

Fertile hyphae branched........................................................................................................................................................................ 24

22(21) Arthroconidia cylindrical; becoming many septate with the age............................................................ **multiseptata**

Arthroconidia barrel-shaped, “T”-shaped, “Y”-shaped, finger-shaped or more irregular, mostly unicellular.............. 23

23(22) Arthroconidia barrel-shaped, 4–8 × 2–3.5 μm; racquet hyphae present...................................................... **chinensis**

Arthroconidia barrel-shaped, “T”-shaped, “Y”-shaped, finger-shaped or more irregular, 2–6 × 1–2 μm; racquet hyphae absent....................................................................................................................................................................... **stricta**

24(21) Fertile hyphae branching acutely, displaying a tree-like appearance.......................................................... **dendritica**

Fertile hyphae branching pattern otherwise.................................................................................................................................... 25

25(24) Fertile hyphae repeatedly branched, in dense tufts...................................................................................... **flocciformis**

Fertile hyphae more restrictedly branched.......................................................................................................................................26

26(25) Colonies buff or tan............................................................................................................................................................. **fulva**

Colonies lemon yellow...................................................................................................................................................................... **flava**

***Key to*** **Spiromastigoides** ***species***

Adapted from Hirooka et al. (2016).

1 Homothallic............................................................................................................................................................................................. 2

Heterothallic................................................................................................................................................................................................ 6

2(1) Ascospores globose to subglobose, reticulate.................................................................................................. **sphaerospora**

Ascospores oblate, equatorial thickening present or not............................................................................................................... 3

3(2) Ascospores with equatorial thickening...................................................................................................................................... 4

Ascospores without such equatorial thickening............................................................................................................................... 5

4(3) Ascomata appendages straight or slightly undulate; ascospores yellow, smooth-walled under LM, pitted under SEM........................................................................................................................................................................ **alatospora**

Ascomata appendages slightly undulate or wavy; ascospores pale yellowish brown, minutely punctate under SEM........................................................................................................................................... **saturnispora**

5(3) Ascospores punctate, sometimes with a few fine grooves in the polar region, 2.5–2.9 × 2.0–2.5 μm...................................................................................................................................................................... **warcupii**

Ascospores lens-shaped, regularly pitted, 3.0 × 2.0 μm................................................................................................ **sugiyamae**

6(1) Asexual morph chrysosporium-like; sterile ascomata present............................................................................. **asexualis**

Asexual morph distinct............................................................................................................................................................................ 7

7(6) Asexual morph malbranchea-like............. .................................................................................................................................. 8

Conidiophores well-developed......... .................................................................................................................................................. 11

8(7) Fertile hyphae straight, branched...................................................................................................................................... **gypsea**

Fertile hyphae curved............................................................................................................................................................................... 9

9(8) Fertile hyphae successively branched to form sporodochia-like structures........................................................... **albida**

Fertile hyphae unbranched or scarcely branched.......................................................................................................................... 10

10(9) Fertile hyphae unbranched or sparsely branched, curved, > to 28 μm long; chlamydospores present...................... **curvata**

Fertile hyphae unbranched, slightly curved, > to 15 μm long; chlamydospores absent......................................... **minimus**

11(7) Conidiophores unbranched or scarcely branched......................................................................................... **geomycoides**

Conidiophores branched several times............................................................................................................................................. 12

12(11) Conidiophores > to 300 μm in length, verticillate............................................................................................ **kosraensis**

Conidiophores 100–150 μm in length, with pyramidal or bush-like branching................................................................... 13

13(12) Conidiophores > to 150 μm long, with pyramidal branching...................................................................*.* **pyramidalis**

Conidiophores > to 100 μm long, with bush-like branching............................................................................................... **frutex**

### IN VITRO ANTIFUNGAL SUSCEPTIBILITY TESTING

The results of the antifungal susceptibility test are summarized in Table 2. In general, the echinocandins (AFG, CFG and MFG) displayed the most potent in vitro antifungal activity, but TRB and PSC also demonstrated a good activity against these fungi. In contrast, limited to no inhibition of growth was observed with AMB, FLC, ITC and 5-FC. Antifungal activity was evaluated against all strains with the exception of FMR 17691, due to the scarce production of conidia and because this strain does not grow in RPMI medium, even after two wk. of incubation.

**Table 2:** Antifungal susceptibility of malbranchea*-*like strains studied

Taxon	Strain	MIC/MEC (μg/mL)									
		AMB	FLC	VRC	ITC	PSC	AFG	CFG	MFG	TRB	5-FC
***Arachnomyces bostrychodes***	FMR 17685	> 16	> 16	2	> 16	> 16	0.03	0.06	0.06	0.5	> 16
***Currahmyces sparsispora***	FMR 17683	> 16	> 16	4	> 16	2	> 16	8	> 16	≤0.03	> 16
***Malbranchea albolutea***	FMR 17679	8	> 16	1	1	0.25	0.03	0.06	0.06	0.25	> 16
	FMR 17689	8	> 16	2	> 16	1	0.12	0.06	0.25	0.25	> 16
***M. aurantiaca***	FMR 17682	> 16	> 16	1	> 16	0.25	0.12	1	0.12	4	> 16
	FMR 17688	> 16	> 16	2	> 16	0.5	0.5	0.06	1	2	> 16
***M. conjugata***	FMR 17697	8	> 16	0.5	0.25	≤0.03	0.06	0.25	0.25	1	> 16
	FMR 17699	> 16	> 16	0.5	2	0.5	0.12	0.25	0.25	1	> 16
***M. flocciformis***	FMR 17698	> 16	> 16	1	> 16	0.5	0.12	0.03	0.12	0,5	> 16
***M. gymnoascoidea***	FMR 17681	> 16	> 16	8	> 16	1	0.03	0.03	0.12	0,5	> 16
***M. multiseptata***	FMR 17695	16	> 16	0.12	0.5	0.25	0.03	0.5	2	1	> 16
***M. stricta***	FMR 17680	8	> 16	0.25	0.12	0.12	0.03	0.25	0.25	0,12	> 16
***M. umbrina***	FMR 17693	4	> 16	2	> 16	0.5	0.06	0.06	0.12	0.25	> 16
	FMR 17694	> 16	> 16	4	> 16	0.5	0.06	1	0.12	0.25	> 16
	FMR 17700	> 16	> 16	> 16	> 16	> 16	0.5	1	0.5	> 16	> 16
	FMR 17701	> 16	> 16	4	> 16	0.12	0.03	0.03	0.03	0.12	> 16
***M. zuffiana***	FMR 17690	> 16	> 16	1	> 16	0.5	0.05	1	4	0.25	> 16
***Pseudomalbranchea gemmata***	FMR 17684	2	> 16	0.25	0.25	0.25	16	1	16	≤0.03	> 16
***Spiromastigoides geomycoides***	FMR 17686	> 16	> 16	2	1	1	> 16	2	> 16	0.12	> 16
	FMR 17696	> 16	> 16	2	0.5	0.5	2	16	> 16	0.06	> 16

*AMB* amphotericin B, *FLC* fluconazole, *VRC* voriconazole, *ITC* itraconazole, *PSC* posaconazole, *AFG* anidulafungin, *CFG* caspofungin, *MFG* micafungin, *TRB* terbinafine, *5-FC* 5-fluorocytosine, *ND* Non-determined due to no fungal growth under the conditions stablished by the CLSI protocol

## DISCUSSION

To our knowledge, this is the main study to be produced on malbranchea-like fungi from a clinical origin to date. We have shown that several of these fungi have not been reported previously from human specimens, and although the pathologic role remains uncertain, their diversity is of interest since some represent new species.

Morphological and physiological characterization and phylogenetic analysis has allowed us to identify 15 strains as belonging to the genus *Malbranchea* (syn. *Auxarthron*), of which three of them are described as new species. These results indicate a high diversity of onygenalean fungi in these sorts of substrates, which may be difficult to differentiate using only phenotypic characteristics.

All strains belonging to *Malbranchea* displayed thermotolerance, suggesting the potential pathogenicity of this genus in animals, including humans, as has been previously noted by others (Saccardo 1908; Saccardo and Trotter 1913; Cooney and Emerson 1964; Sigler and Carmichael 1976). All were able to grow at 30 °C, and most of them at 35–37 °C.

Malbranchea-like fungi were most commonly isolated from the respiratory tract (40%) followed by nails and skin (27.2%). *Currahmyces sparsispora*, *Malbranchea albolutea*, *M. conjugata*, *M. gymnoascoides*, *M. multiseptata*, *Pseudoarthropsis crassispora,* and *Pseudomalbranchea gemmata* were all recovered from respiratory tract specimens (mostly obtained by bronchial-alveolar washing), while those of *M. umbrina* were isolated from the widest variety of anatomical sites. The rest of the taxa isolated were mostly from skin and annexes.

Regarding the antifungal susceptibility of malbranchea*-*like fungi, limited data are available. However, in a previous study on onychomycosis-causing strains of *Auxarthron ostraviense* and *Auxarthron umbrinum* (transferred to *Malbranchea* in the present study) reduced susceptibility to AMB, ITC and PSC was reported, but a high susceptibility to TRB was observed (Hubka et al. 2013). Another study (Gupta and Kohli 2003) showed that strains of *Arachnomyces nodosetosus* (syn. *Onychocola canadensis*) where highly susceptible to ciclopirox and TRB. Our results are consistent with such previous studies, but we also demonstrated the enhanced susceptibility of the malbranchea*-*like fungi to the echinocandins.

## CONCLUSIONS

From all malbranchea-like strains from clinical specimens (mostly human) in the USA that we studied, only 13 out of 22 could be identified at the species level, three of them belonging to the genus *Malbranchea.* With the exception of one strain initially identified as *Currahmyces indicus*, the others were identified as species of *Auxarthron*, a genus synonymized with *Malbranchea* during the course of the present work. Eight of the remaining strains have been assimilated to the genera *Arachnomyces* (2), *Arthropsis* (1), *Malbranchea* (3), and *Spiromastigoides (*2), the latter only located at family level (Onygenaceae). This is an extraordinary finding, because nearly half of the fungal strains presumed to belong to the genus *Malbranchea* resulted in becoming new taxa for science. Finally, despite the lack of histopathological data, which could have undoubtedly proven that these strains were the causative agents of the infections that led to the request for sample collection, we would highlight their poor sensitivity to first-line drugs such as AMB, FLC, and ITC, but better sensitivity to echinocandins and PSC.

## Supplementary Information

**Additional file 1 **: **Fig. S1.** ML phylogenetic tree based on the analysis of ITS nucleotide sequences for the 22 clinical fungi from the USA. Bootstrap support values/Bayesian posterior probability scores of 70/0.95 and higher are indicated on the nodes. ^T^ = ex type. Fully supported branched (100% BS /1 PP) are indicated in bold. Strains identified by us are in bold. *Arachnomyces* spp. were chosen as out-group. The sequences used in this analysis are in Table 1.

**Additional file 2 **: **Figure S2.** ML phylogenetic tree based on the analysis of LSU nucleotide sequences for the 22 clinical fungi from the USA. Bootstrap support values/Bayesian posterior probability scores of 70/0.95 and higher are indicated on the nodes. ^T^ = ex type. Fully supported branched (100% BS /1 PP) are indicated in bold. Strains identified by us are in bold. *Arachnomyces* spp. were chosen as out-group. The sequences used in this analysis are in Table 1.

## Data Availability

All data generated or analysed during this study are included in this published article.

## ABBREVIATIONS

5-FC: 5-fluorocytosine
AFG: Anidulafungin
AMB: Amphotericin B
BCP-MS-G: Bromocresol purple milk solids glucose agar
BI: Bayesian-inference
BLAST: Basic Local Alignment Search Tool
BS: Bootstrap support
CFG: Caspofungin
CLSI: Clinical and Laboratory Standards Institute
DNA: Deoxyribonucleic acid
FLC: Fluconazole
ITC: Itraconazole
ITS: Ribosomal internal transcribed spacers
LM: Light microscope
LSU: Large sub unit of the ribosomal genes
MEC: Minimal Effective Concentrations
MFG: Micafungin
MIC: Minimal Inhibitory Concentrations
ML: Maximum-likelihood
MLI: Maximum level of identity
OA: Oatmeal agar
PDA: Potato dextrose agar
PP: Posterior probability
PSC: Posaconazole
PYE: Phytone yeast extract agar
SDA: Sabouraud dextrose agar
SEM: Scanning electron microscopy
TOTM: Test opacity tween medium
TRB: Terbinafine
TreeBASE: A repository of user-submitted phylogenetic trees and data used to build them
USA: United States of America
UTHSCA: University of Texas Health Science Centre at San Antonio
VRC: Voriconazole
