# Supplementary figures and images for: A revision of malbranchea-like fungi from clinical specimens in the United States of America reveals unexpected novelty
Source: IMA Fungus. 2021 Sep 7;12:25. doi: 10.1186/s43008-021-00075-x (PMC8422767; doi:10.1186/s43008-021-00075-x)

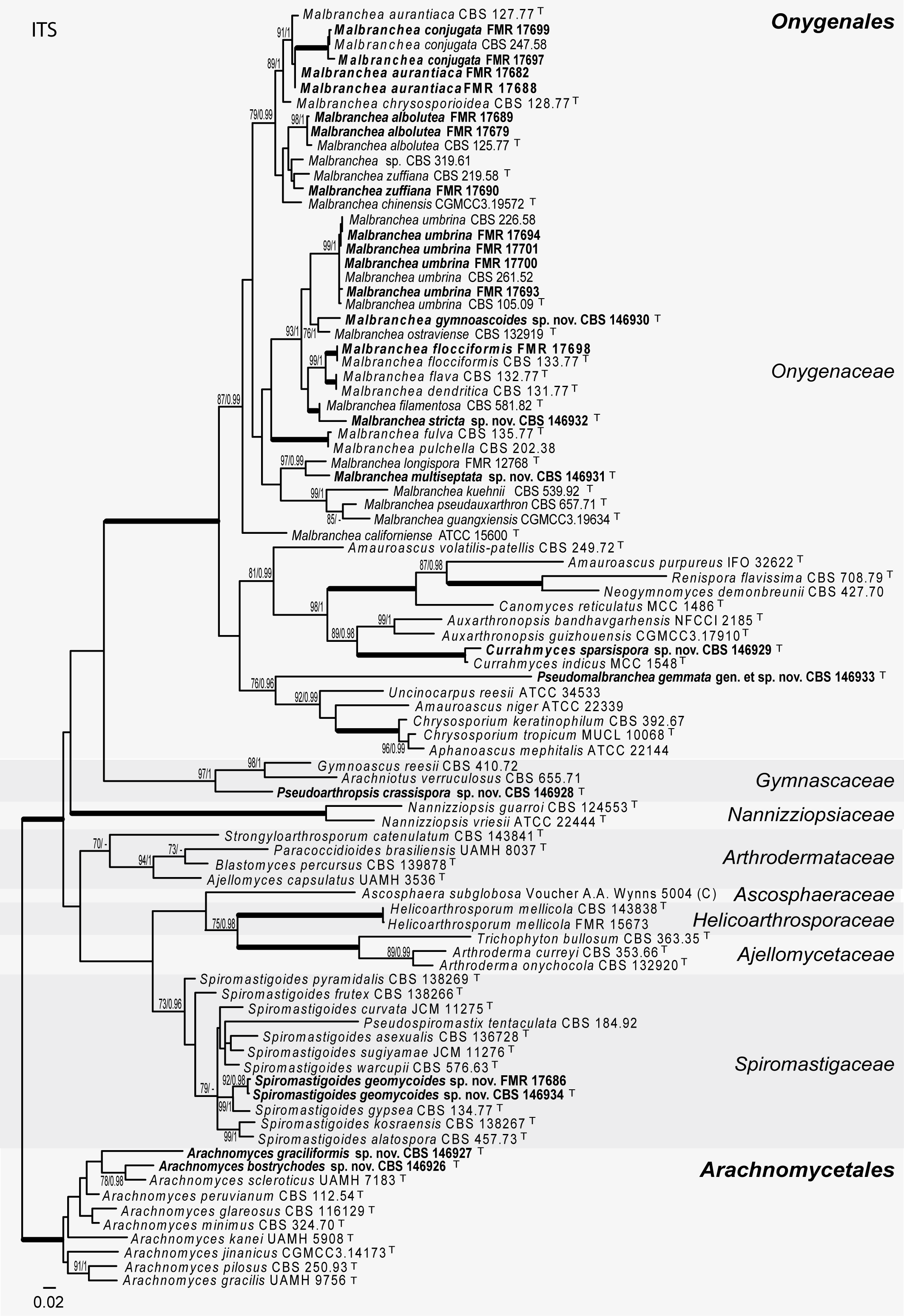

Supplement: Supplementary file 1 — Additional file 1 : Fig. S1. ML phylogenetic tree based on the analysis of ITS nucleotide sequences for the 22 clinical fungi from the USA. Bootstrap support values/Bayesian posterior probability scores of 70/0.95 and higher are indicated on the nodes. T = ex type. Fully supported branched (100% BS /1 PP) are indicated in bold. Strains identified by us are in bold. Arachnomyces spp. were chosen as out-group. The sequences used in this analysis are in Table 1. [file 43008_2021_75_MOESM1_ESM.tif]

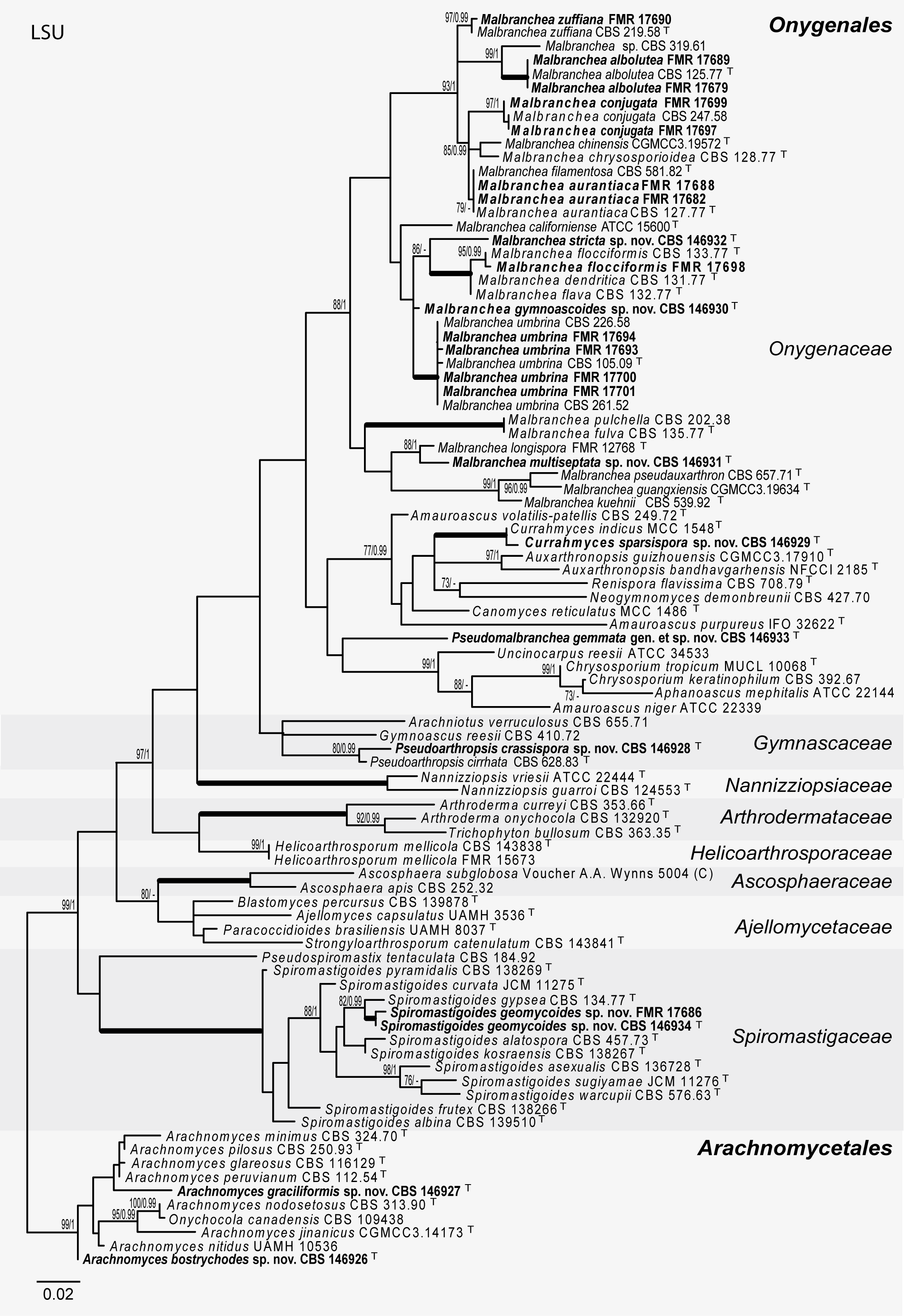

Supplement: Supplementary file 2 — Additional file 2 : Figure S2. ML phylogenetic tree based on the analysis of LSU nucleotide sequences for the 22 clinical fungi from the USA. Bootstrap support values/Bayesian posterior probability scores of 70/0.95 and higher are indicated on the nodes. T = ex type. Fully supported branched (100% BS /1 PP) are indicated in bold. Strains identified by us are in bold. Arachnomyces spp. were chosen as out-group. The sequences used in this analysis are in Table 1. [file 43008_2021_75_MOESM2_ESM.tif]
